# Supplementary material for: Toward a base-resolution panorama of the in vivo impact of cytosine methylation on transcription factor binding
Source: Genome Biol. 2022 Jul 7;23:151. doi: 10.1186/s13059-022-02713-y (PMC9264634; doi:10.1186/s13059-022-02713-y)
Supplement: Supplementary file 1 — Additional file 1: Fig S1-18. Figure S1. JAMS sequence coefficients for CTCF in HEK293 cells. Figure S2. TF-specific and background coefficients for CTCF in HEK293 cells. Figure S3. Likelihood ratio test per position to identify CTCF binding site positions with significant methylation effects. Figure S4. JAMS coefficients for CTCF across different cell lines. Figure S5. Calculating logFC S.E.M. threshold. Figure S6. Predicting differential CTCF binding independent of regional methylation. Figure S7. JAMS results by TF families. Figure S8. Example high-quality and low-quality JAMS models. Figure S9. Annotation of the zinc finger domains whose binding to DNA are affected by CpG methylation. Figure S10. In vivo methylation binding preferences of CEBBP and NFR1. Figure S11. Predicting differential binding of CEBPB across cell lines. Figure S12. Predicting differential binding of MAX across cell lines. Figure S13. Predicting differential binding of KAISO (ZBTB33) across cell lines. Figure S14. Comparison of methyl-sensitive positions identified by JAMS and bisulfite-SELEX. Figure S15. Methyl-plus and mixed-effect TFs identified by JAMS. Figure S16. Modeling choices for analysis of CTCF occupancy in HEK293 cells. Figure S17. Effect of mC12pG13 methylation on in vivo CTCF binding. Figure S18. Effect of mC12pG13 methylation on in vitro CTCF binding. [file 13059_2022_2713_MOESM1_ESM.pdf]

Supplementary Figures for

Toward a base-resolution panorama of the *in vivo* impact of cytosine methylation on transcription factor binding

Aldo Hernandez-Corchado[1,2], Hamed S. Najafabadi[1,2,]*

[1] Department of Human Genetics, McGill University, Montreal, QC H3A 1B1, Canada
[2] McGill Genome Centre, Montreal, QC H3A 0G1, Canada

* Corresponding author: H. S. Najafabadi, hamed.najafabadi@mcgill.ca

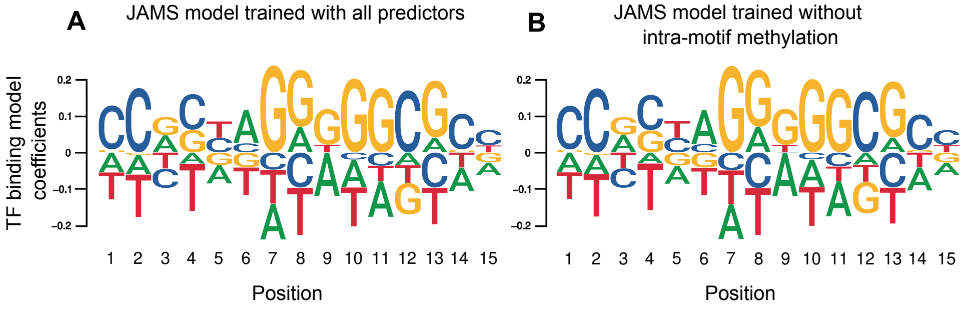

Fig S1. JAMS sequence coefficients for CTCF in HEK293 cells. (**A**) Sequence logo representation of the coefficients of a JAMS model trained using all variables. (**B**) Same as panel A, but for a JAMS model that was trained after excluding intra-motif CpG methylation variables, showing that inference of CTCF sequence specificity is robust to the inclusion/exclusion of methylation status.

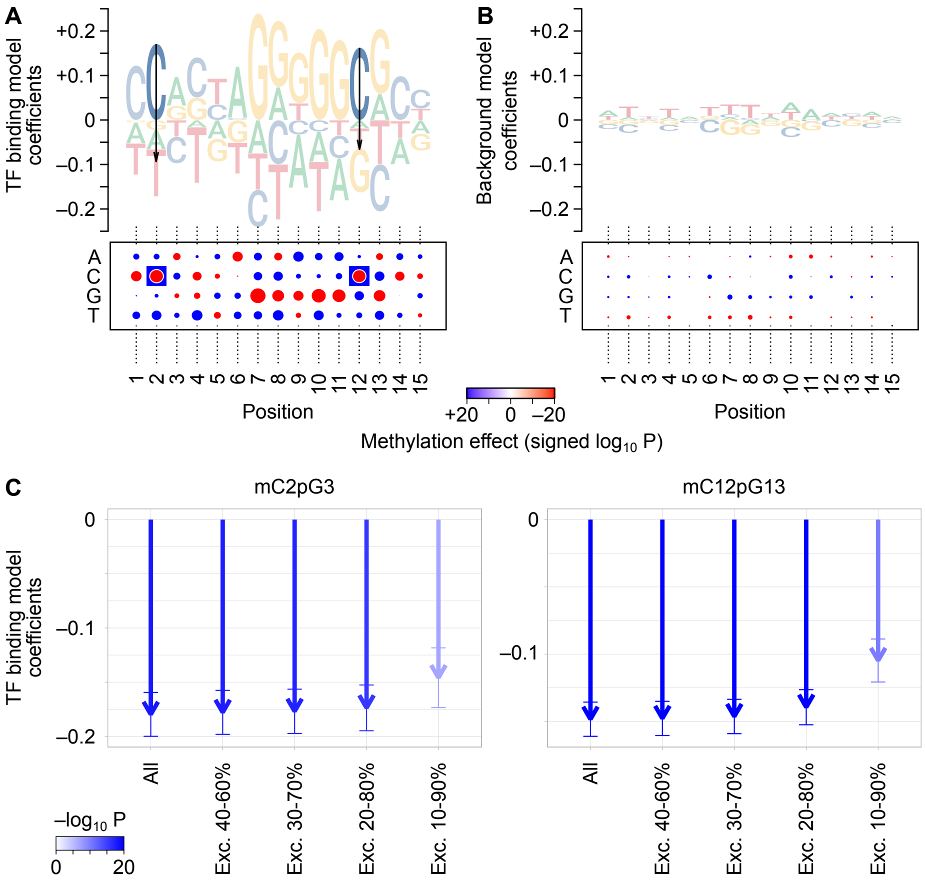

Fig S2. TF-specific and background coefficients for CTCF in HEK293 cells. (**A**) Motif logo and dot plot representations of the sequence/methylation preference of the TF-specific signal. The logo (top) shows methylation coefficients as arrows, with the arrow length proportional to the mean estimate of methylation effect. The heatmap (bottom) shows the magnitude of the preference for each nucleotide at each position using the size of the dots, with red and blue representing positive and negative coefficients, respectively. The signed logarithm of P-value of the methylation coefficient is shown using the color of the squares around the dots, with red and blue corresponding to increased or decreased binding to methylated CpG, respectively (only significant methylation coefficients at FDR<1×10–5 are shown). Note that while the methylation coefficient corresponds to the entire CpG dinucleotide, only the C in the CpG dinucleotide is marked with the colored squares. (**B**) Motif logo and dot plot representations for the background signal. (**C**) Robustness of JAMS methylation coefficients against the presence of intermediate methylation states for positions C2pG3 (left) and C12pG13 (right). Arrows show the estimated coefficient of methylation obtained using all peaks (“All”), or after excluding peaks with intermediate methylation levels, with the intermediate range progressively increasing from 40-60% to 10-90% (and, therefore, progressively fewer peaks are kept for model fitting). The color of the arrow shows the logarithm of the P-value of the methylation coefficient, and error bars show the standard error.

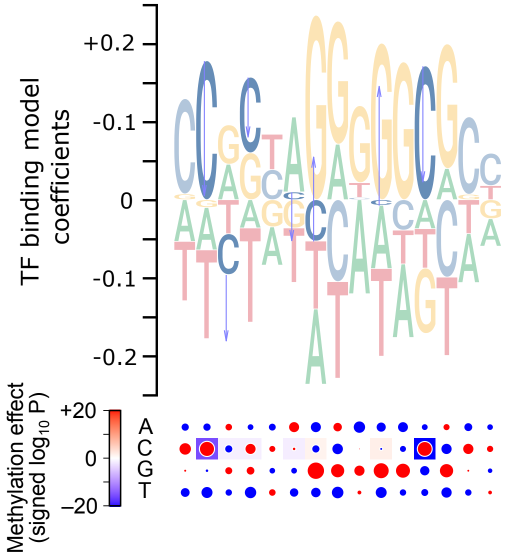

Fig S3. Likelihood ratio test per position to identify CTCF binding site positions with significant methylation effects. For each position of the binding site, a reduced model was trained, each exclusing methylation of that position from the predictive variables. Then, each of these reduced models were compared to the whole CTCF JAMS model using a likelihood ratio test (LRT). The p-values obtained from the LRT are shown as the color of the squares. The effect sizes for the bases and methylation are obtained from the full CTCF JAMS model. Significant LRT p-values indicate that removing the methylation of the corresponding position from the model reduces the goodness of fit. The motif logo and dot plot representations follow the same notations as **Fig S2**.

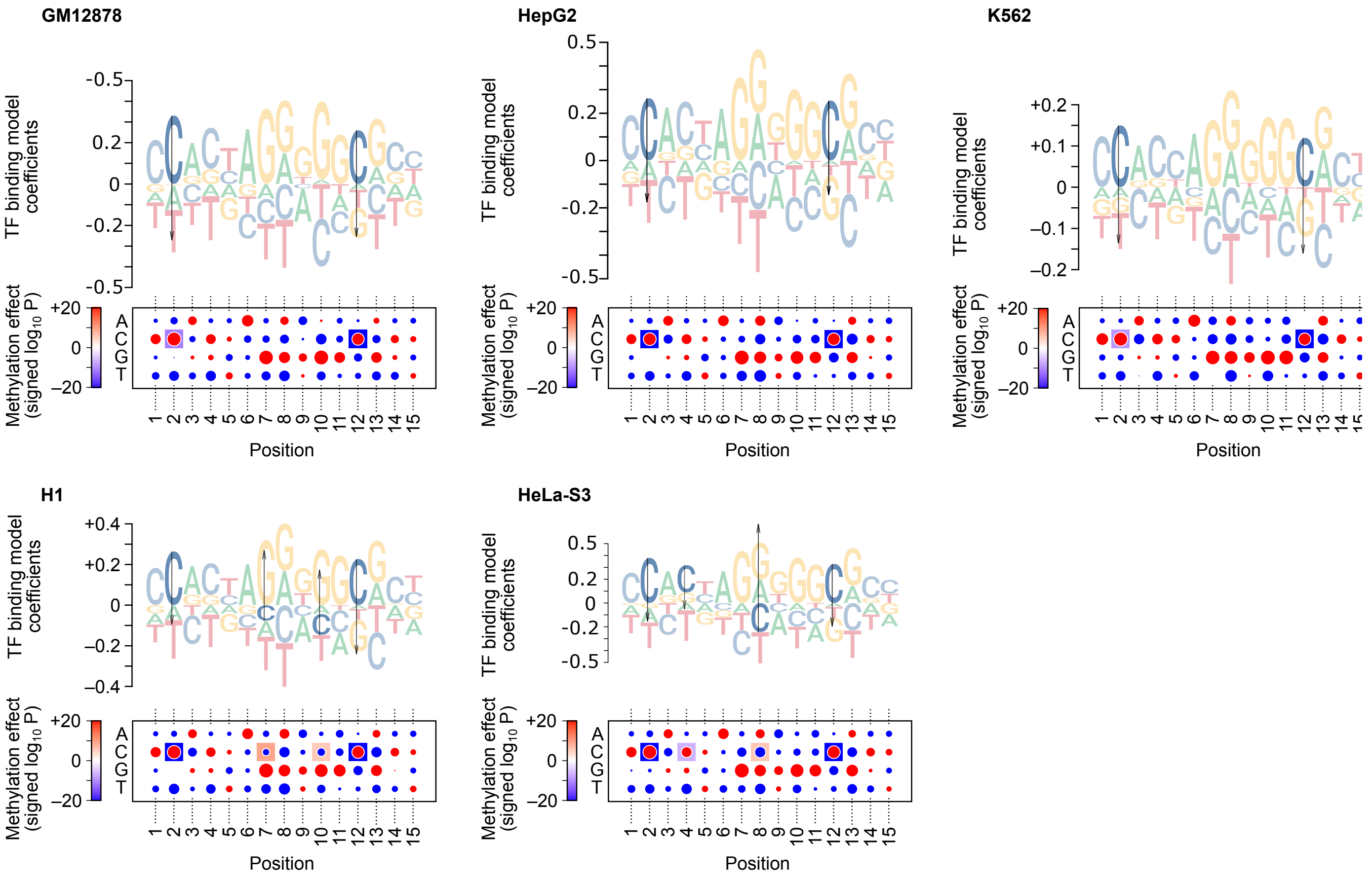

Fig S4. JAMS coefficients for CTCF across different cell lines. Motif logs and dot plot representations follow the same format as described in **Fig S2**. TF-specific and background model coefficients are shown side-by-side for each of the six cell lines (GM12878, HepG2, H1, HeLa-S3, and K562).

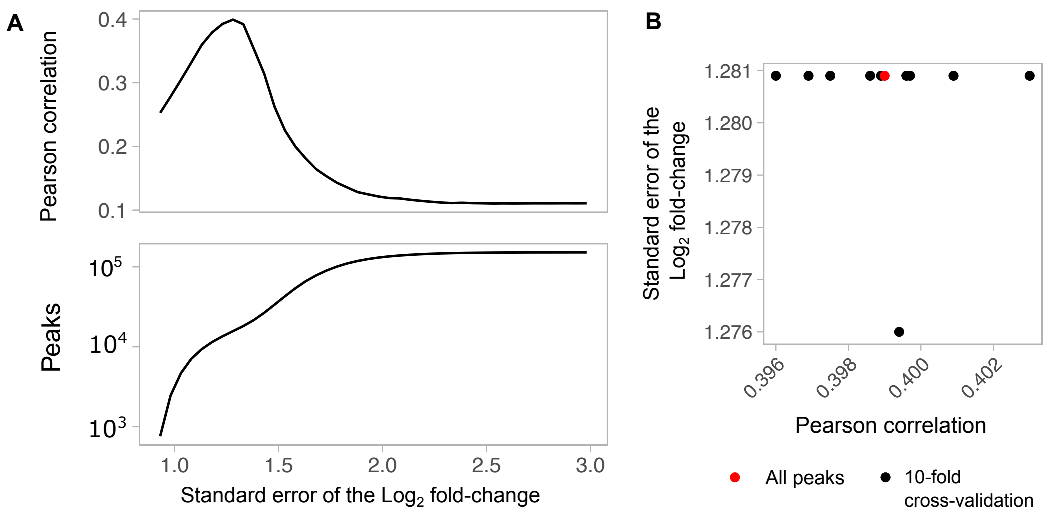

Fig S5. Calculating logFC S.E.M. threshold. (**A**) Pearson correlation between the predicted and observed change in CTCF binding, after filtering the CTCF peaks based on different cutoffs for standard error of mean (S.E.M.) of the LFC of pull-down/control ratio. An optimal threshold is observed at logFC S.E.M. = 1.28. (**B**) To discard the possibility of overfitting of the threshold, different optimal thresholds were calculated using a 10-fold cross-validation approach. Specifically, each time 90% of the peaks were used to identify the optimal logFC S.E.M threshold, and the Pearson correlation between the predicted and observed peaks that passed that threshold was calculated on the remaining 10% of peaks The logFC S.E.M. threshold obtained by using all peaks (red dot) is similar to the thresholds obtained with cross-validation (black dots), and leads to a similar correlation between the predicted and observed change in CTCF binding for the held-out peak sets (ranging from 0.396 to 0.403).

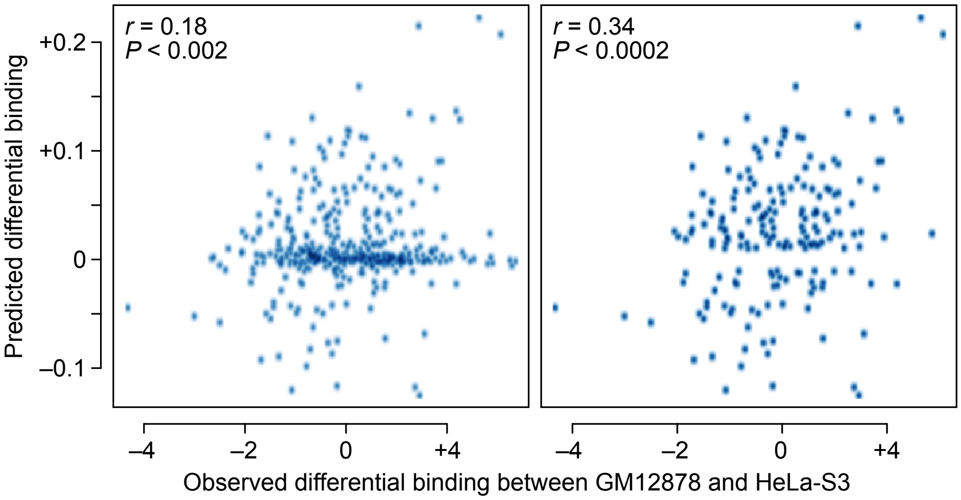

Fig S6. Predicting differential CTCF binding independent of regional methylation. The scatterplots show the JAMS-predicted changes in CTCF binding (y-axis) vs. the observed differential binding between GM12878 and HeLa-S3 cells. Only the peaks that have no CpGs in the binding site flanking regions (20bp) and have no-change in accessibility between the two cell lines (difference in accessibility < 0.2) are included in the left scatterplot. In addition to these filters, in the right scatterplot, the peaks that have no differential intra-motif methylation (i.e. have no position with |Δmethylation| > 0.05) are removed.

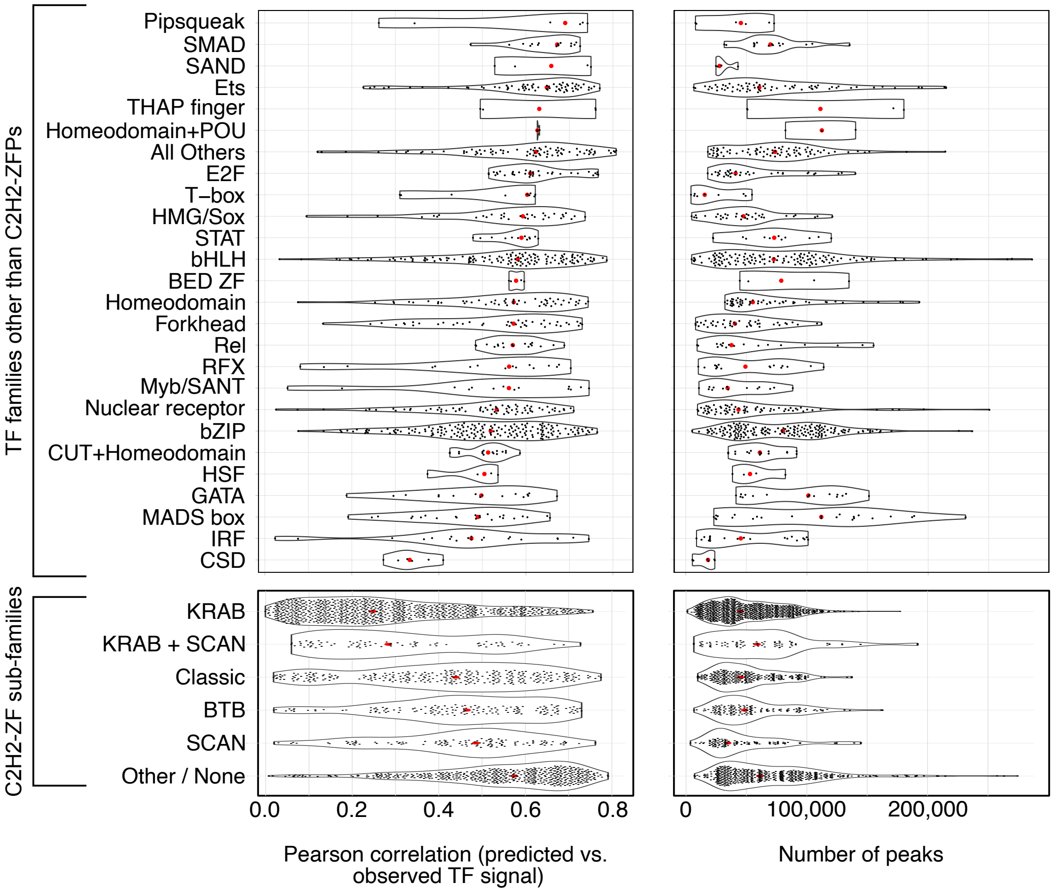

Fig S7. JAMS results by TF families. Violin plots showing the Pearson correlation between observed and predicted pull-down tag density (left) and number of peaks used to train the GLM (right), shown separately for each TF family (top) and C2H2-ZF subfamilies (bottom).

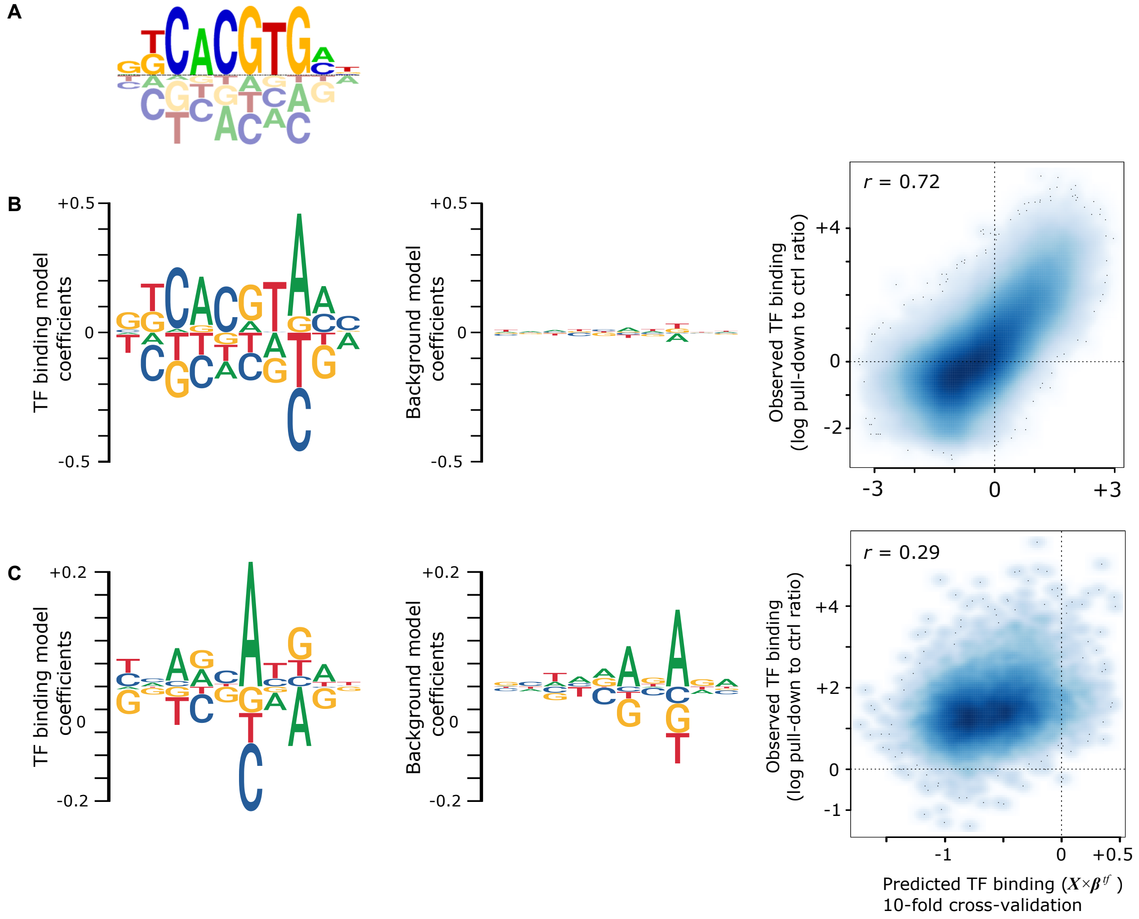

Fig S8. Example high-quality and low-quality JAMS models. (**A**) The known BHLHE40 motif, obtained from the CIS-BP database, shown as an example (motif ID M02788_2.00). (**B-C**) Results from a high-quality (**B**) and a low-quality (**C**) JAMS model for BHLHE40. Inferred sequence coefficients for TF binding (left) and background (middle), as well as the predicted vs. observed TF binding signal (right) are shown.

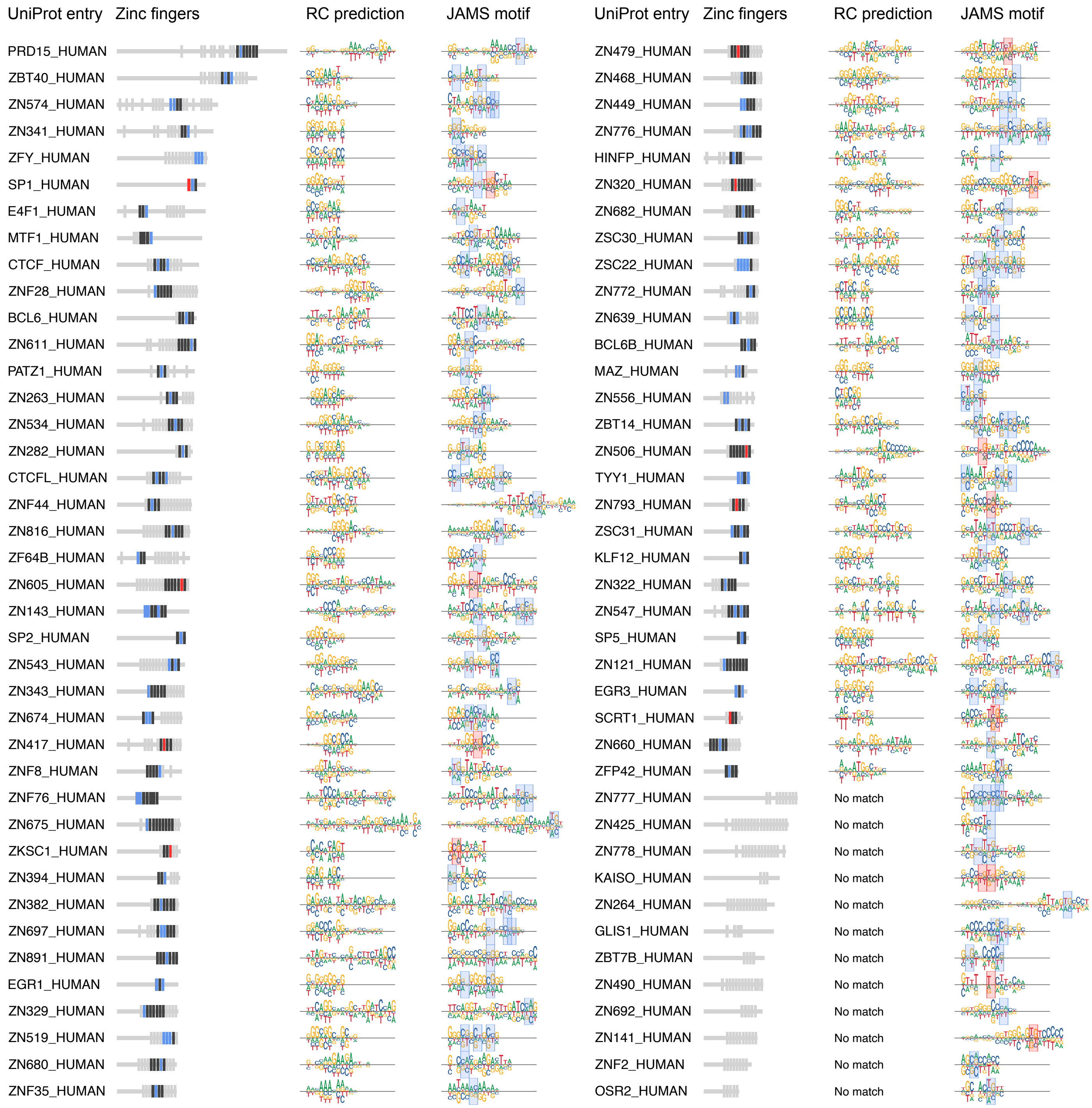

Fig S9. Annotation of the zinc finger domains whose binding to DNA are affected by CpG methylation. For each C2H2-ZF protein, the ZF domain organization is schematically shown, followed by the motif that is predicted from protein sequence using a random forest-based recognition code (RC) [46], as well as the motif obtained by JAMS. The RC motif was used as a guide in order to align the JAMS motif to the zinc finger domains. Methyl-minus and methyl-plus positions in the JAMS motifs are shown with blue and red boxes, respectively, and the corresponding zinc fingers are colored accordingly.

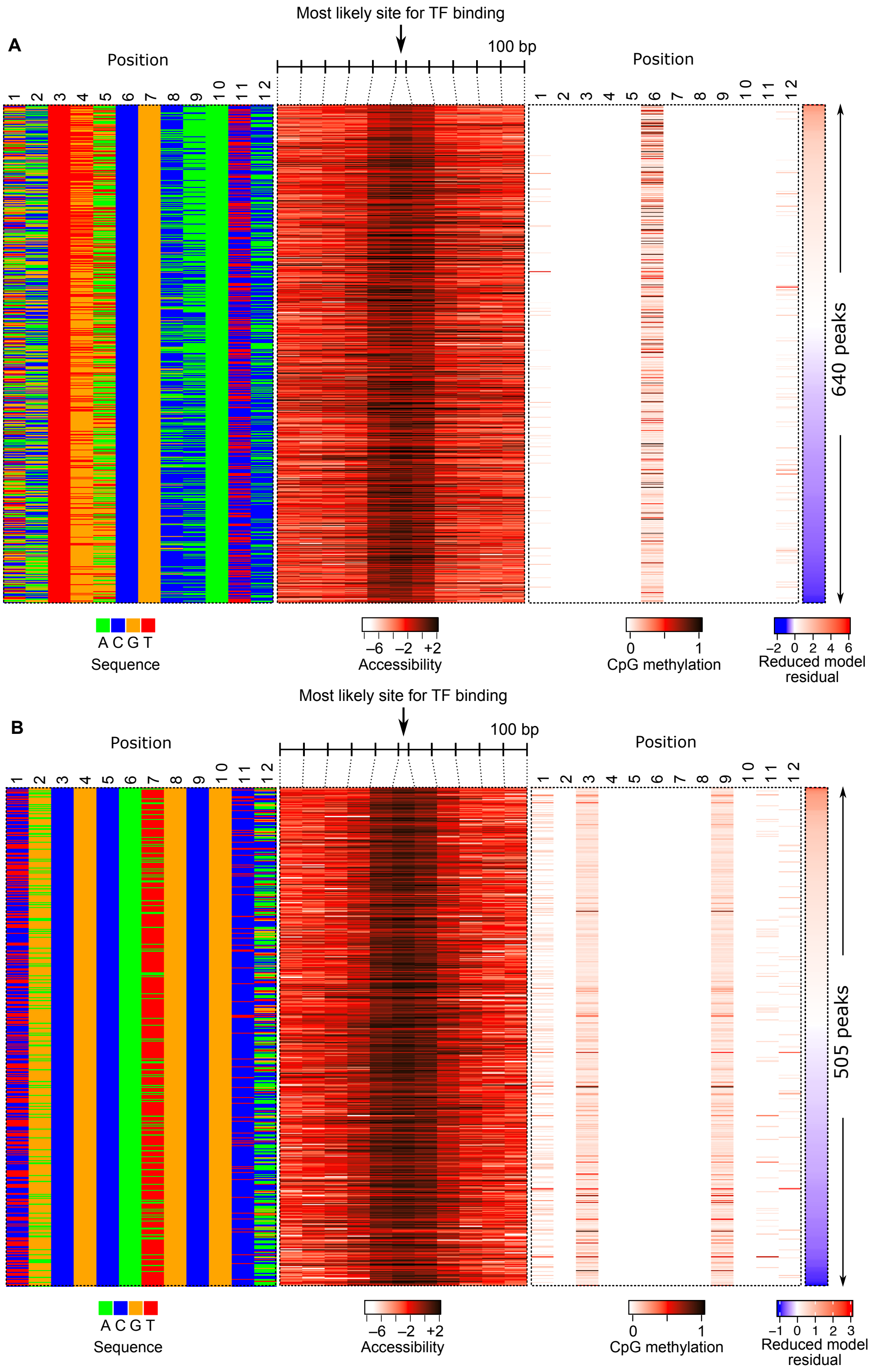

Fig S10. *In vivo* methylation binding preferences of CEBBP and NFR1. (**A**) Heatmap representation of the sequence, accessibility, and CpG methylation, for a subset of CEBPB peaks that have high DNA accessibility, are similar to the CEBPB consensus binding sequence, and have a CpG in position 6/7. Peaks are sorted by the residual of a reduced JAMS model that does not use the methylation level for predicting the TF binding signal. (**B**) Same as panel A, but for NFR1 (with the requirement to have CpG dinucleotides at positions 3/4 and 9/10).

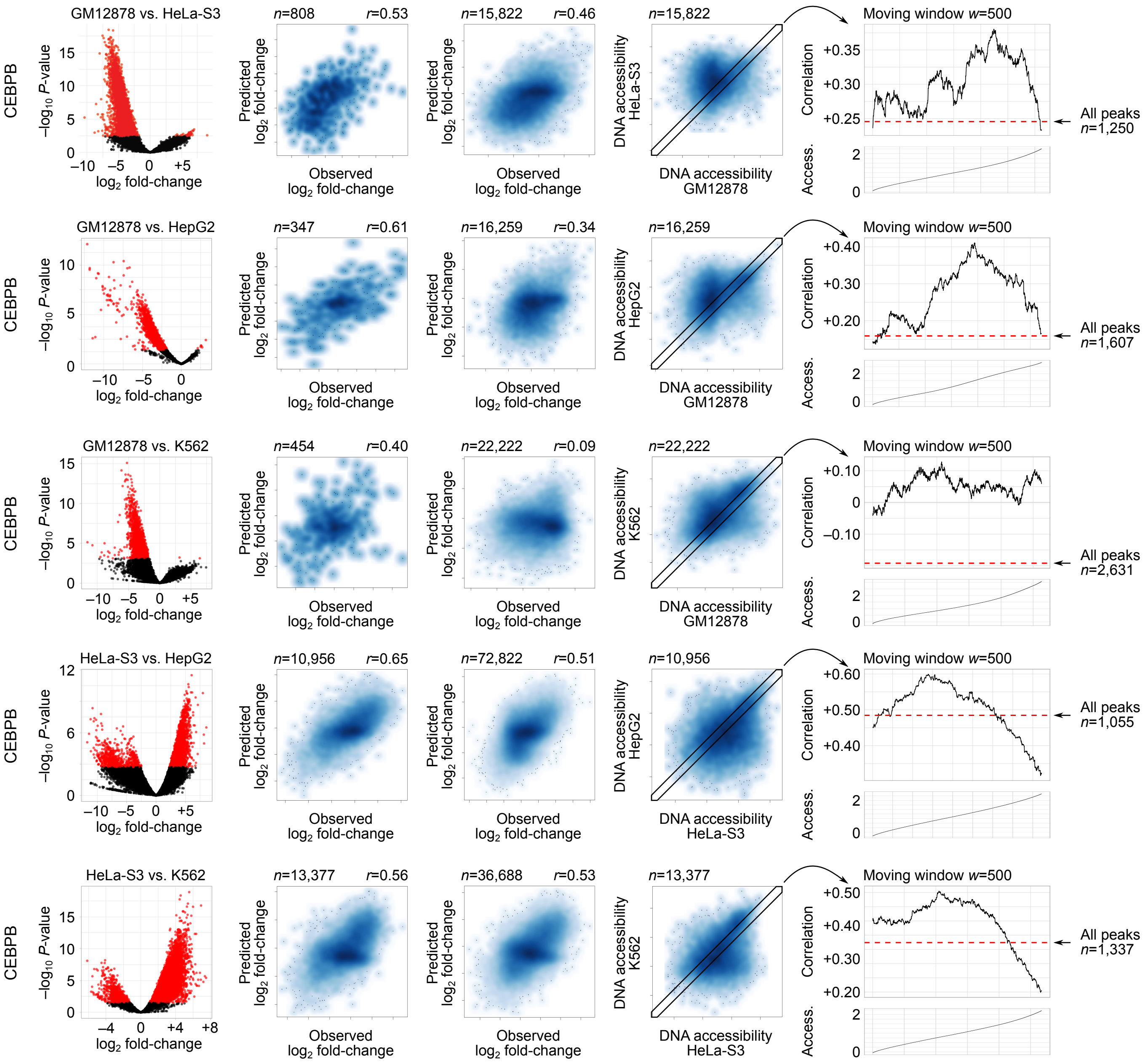

Fig S11. Predicting differential binding of CEBPB across cell lines. Each rows represents differential binding analysis in one pair of cell lines. From left to right for each row: Volcano plot showing the differential binding analysis results; The scatterplot of JAMS-predicted vs. observed differential binding for a set of peaks with a stringent logFC SEM threshold chosen similar to **Fig S5**; The scatterplot of JAMS-predicted vs. observed differential binding for peaks that pass the default logFC SEM threshold <1; Comparison of the accessibility of putative peaks between two cell lines; The correlation between JAMS-predicted and observed differential binding for peaks with no change in accessibility between the two cell lines, similar to **Fig 3E**.

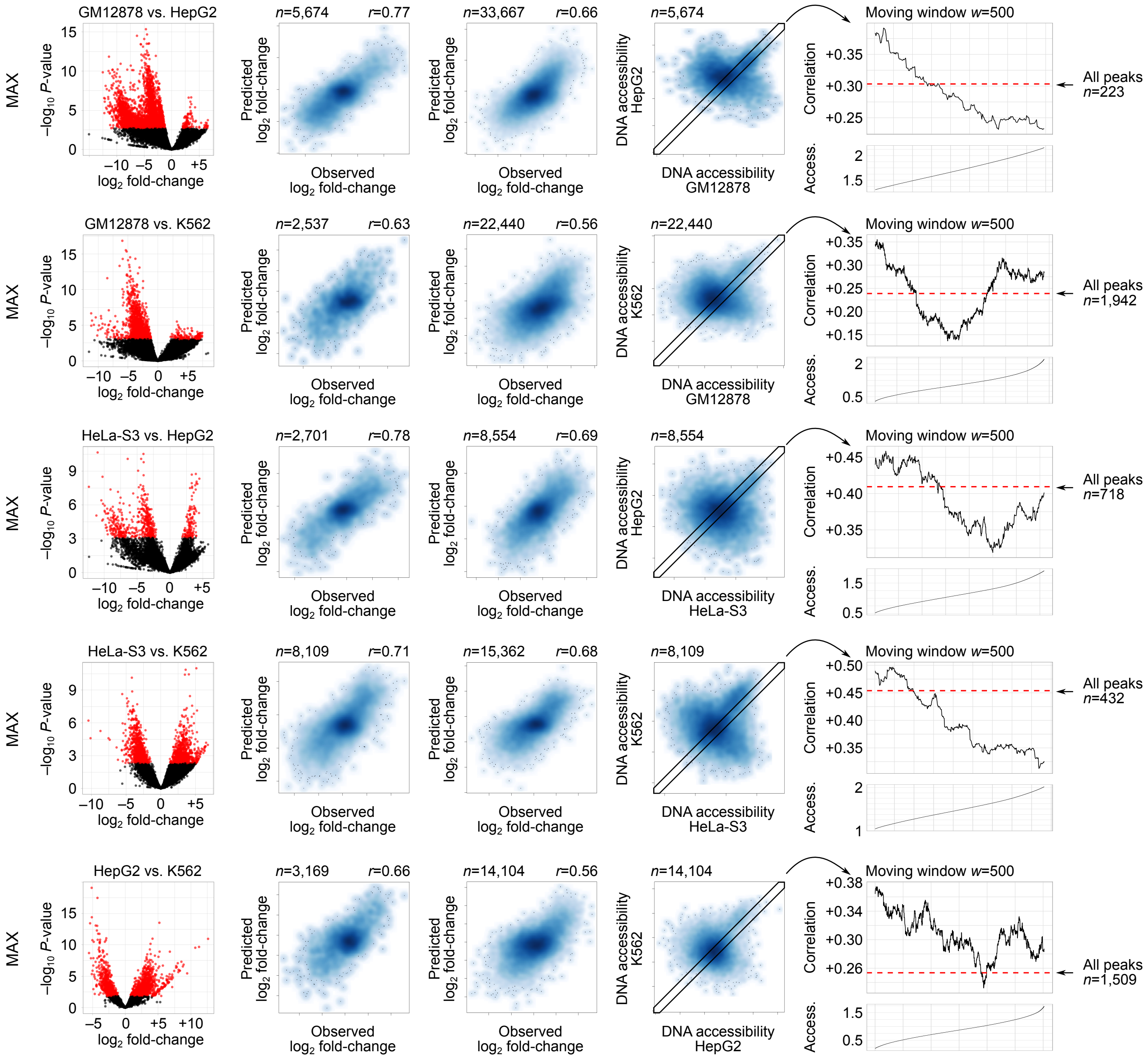

Fig S12. Predicting differential binding of MAX across cell lines. See **Fig S11** for a description of the panels.

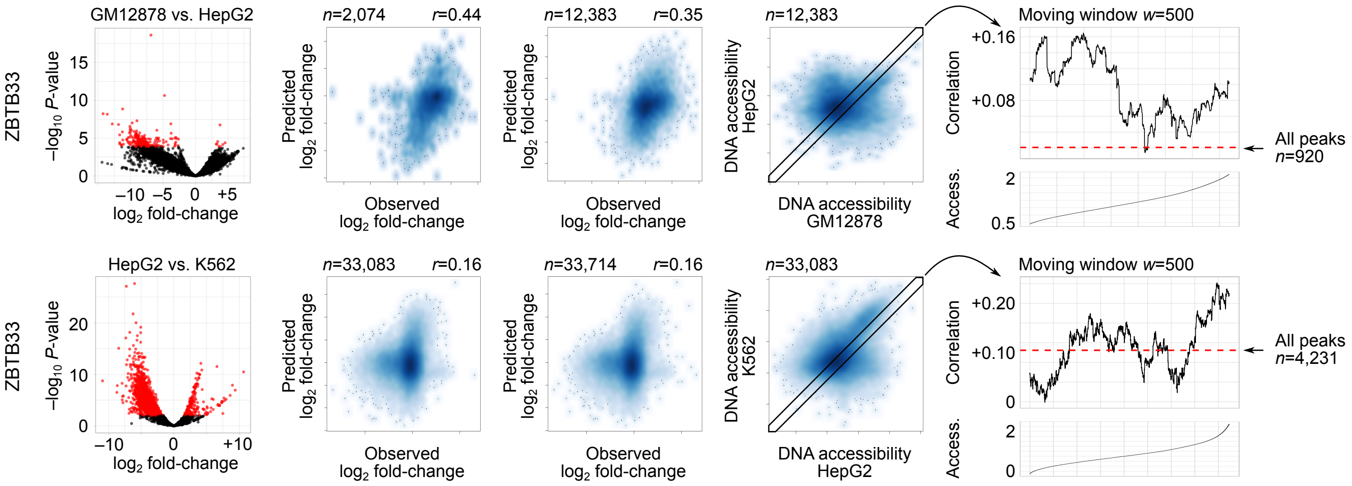

Fig S13. Predicting differential binding of KAISO (ZBTB33) across cell lines. See **Fig S11** for a description of the panels.

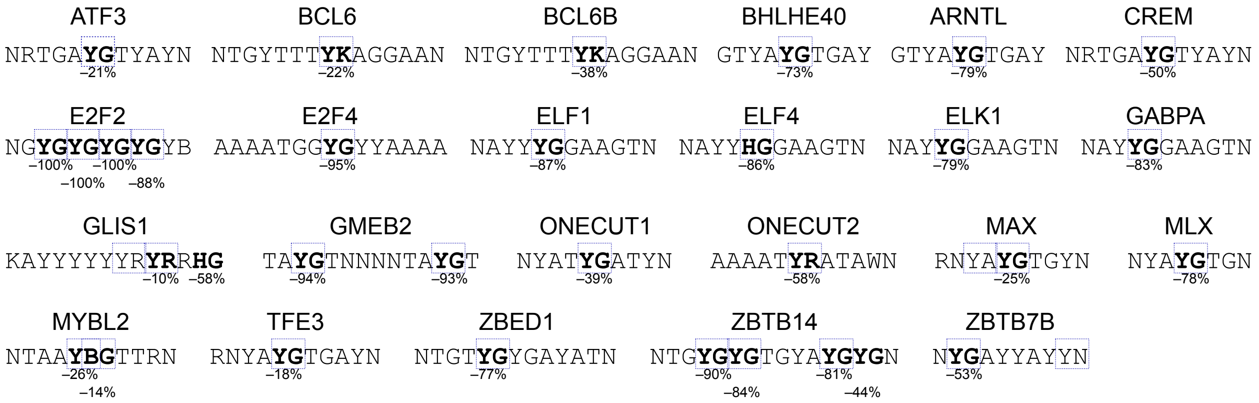

Fig S14. Comparison of methyl-sensitive positions identified by JAMS and bisulfite-SELEX. Only TFs that are identified by both JAMS and bisulfite-SELEX [5] as methyl-minus are included. For each TF, the seed sequence identified by Yin et al. using bisulfite-SELEX analysis [5] is shown using IUPAC nucleotide symbols. The positions whose methylation is found by bisulfite-SELEX to affect TF binding are highlighted using bold font, and the mCpG depletion percentage is shown below the dinucleotide. The positions that are identified by JAMS are shown with a box around the corresponding dinucleotides.

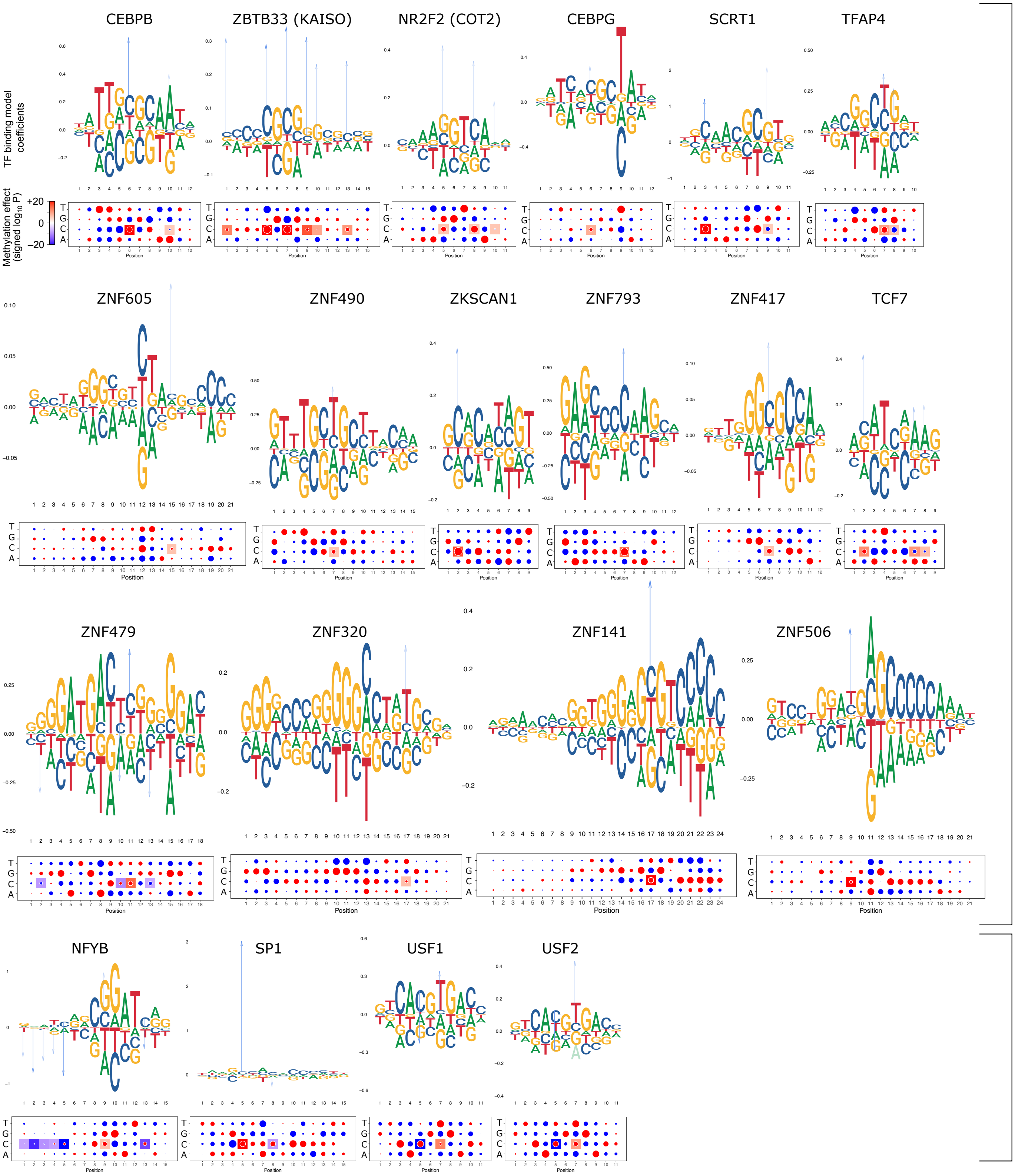

Fig S15. Methyl-plus and mixed-effect TFs identified by JAMS. Motif logo and dot plot representations follow the same formatting as **Fig S2**.

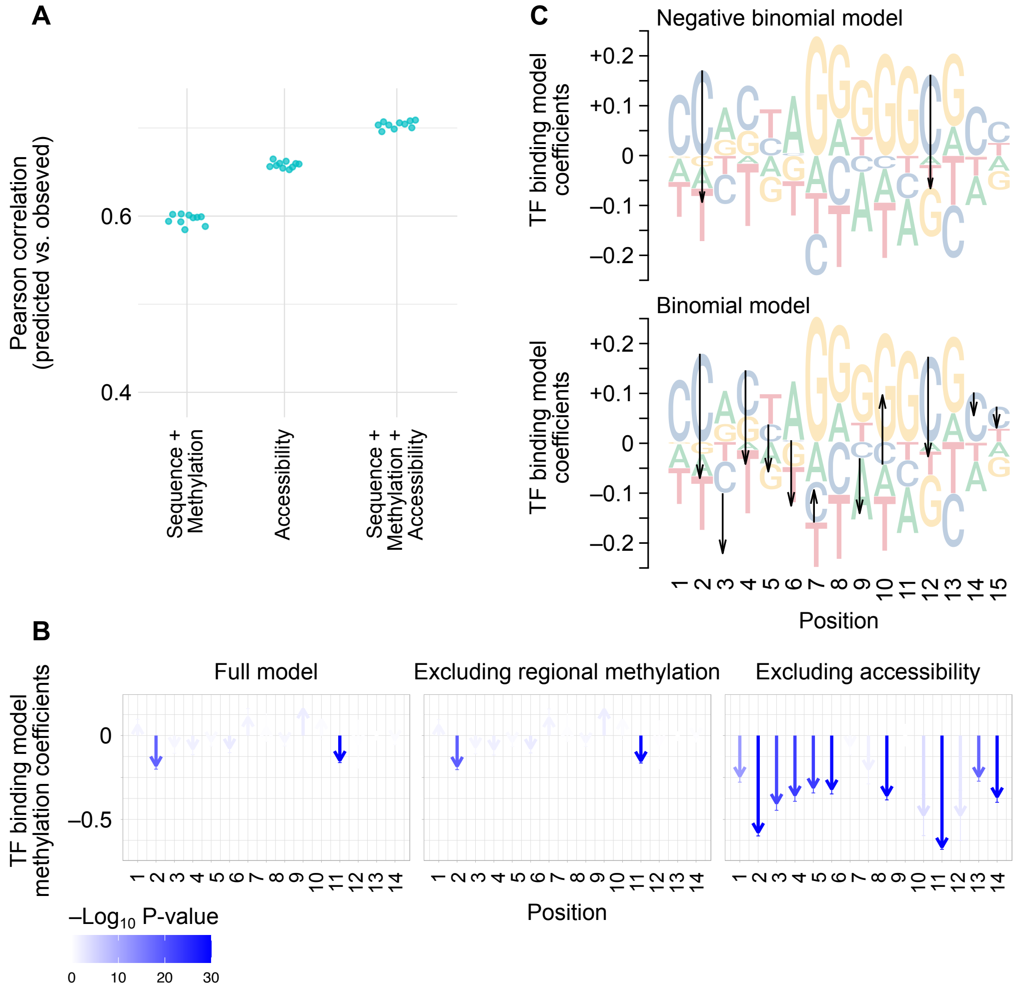

Fig S16. Modeling choices for analysis of CTCF occupancy in HEK293 cells. (**A**) We trained separate JAMS models for CTCF while considering only sequence and methylation features within the motif and flanking regions, only accessibility within and around the motif, or the combination of accessibility/methylation/sequence features. In each case, we performed 10-fold cross-validation; the figure shows the predicted vs. observed pulldown-to-control ratio for each of these scenarios in the 10-fold cross-validation experiment. (**B**) Intra-motif methylation coefficients for JAMS models trained using all variables (left), all variables except regional (flanking) methylation (centre), or all variables except DNA accessibility (right). Arrows show the estimated coefficient of methylation per position. The color of the arrow shows the logarithm of the P-value of the methylation coefficient, and error bars show the standard error. (**C**) The top logo shows the sequence and methylation features identified by JAMS when a negative binomial error model is used (same as **Fig. 2A**). The bottom logo shows the results from a model based on the approach used by [34], in which the pulldown-to-control ratio is modeled as a function of the dependent variables using GLM with binomial family. Arrows correspond to methylation effects that are significant at FDR<10^{-5}.

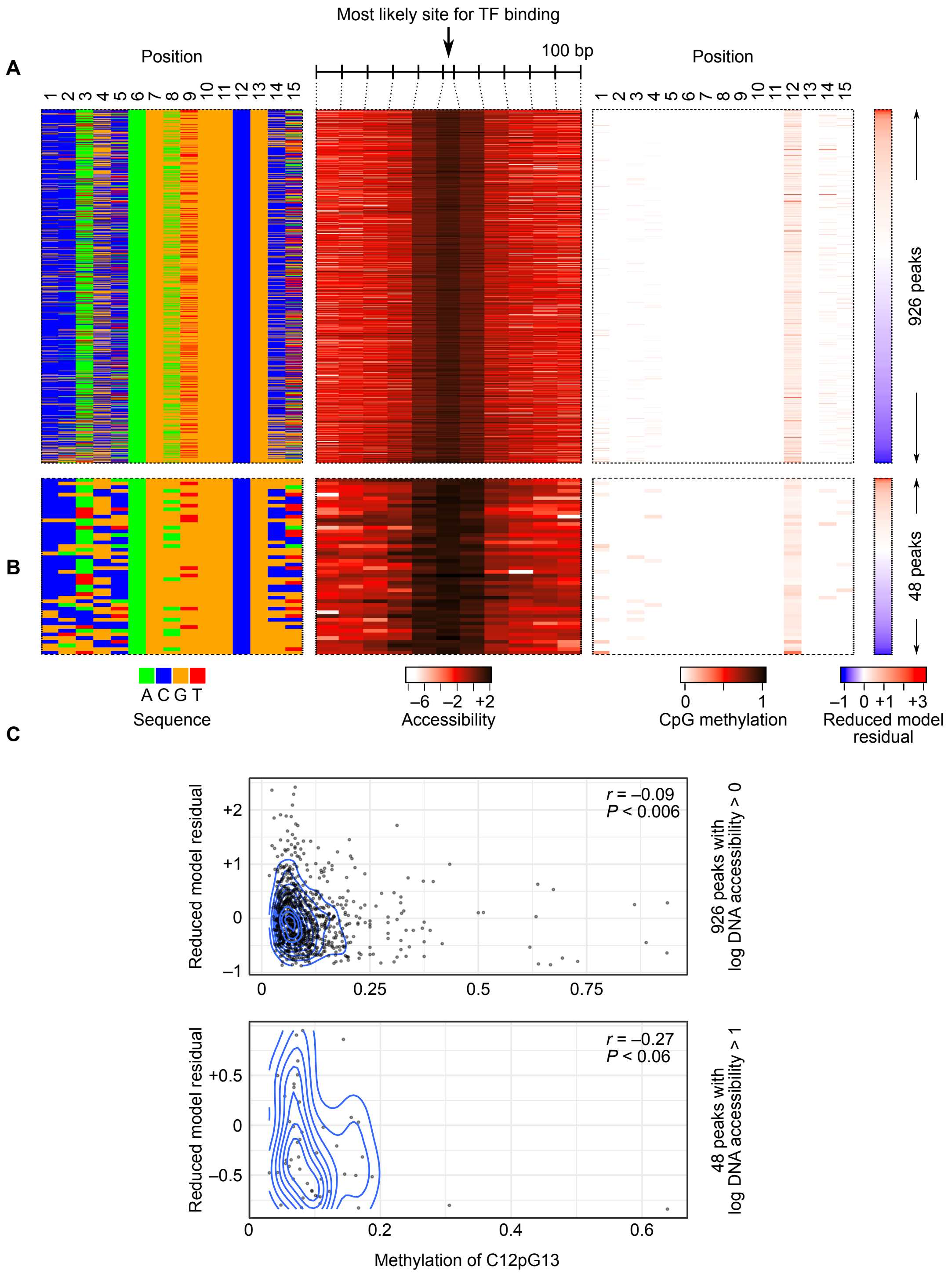

Fig S17. Effect of mC12pG13 methylation on *in vivo* CTCF binding. (**A**) Heatmap representation of the sequence, accessibility, and CpG methylation, for a subset of CTCF peaks that have high DNA accessibility (log-normalized signal >0), are similar to the CTCF consensus binding sequence, and have a CpG in position 12/13, and lack a CpG in position 2/3. Peaks are sorted by the residual of a reduced JAMS model that does not use the methylation level for predicting the TF binding signal. (**B**) Similar to panel A, but using a more stringent accessibility threshold (log-normalized signal >1). (**C**) Scatterplot of the relationship between mC12pG13 and reduced JAMS model residuals from panel A (top) or panel B (bottom).

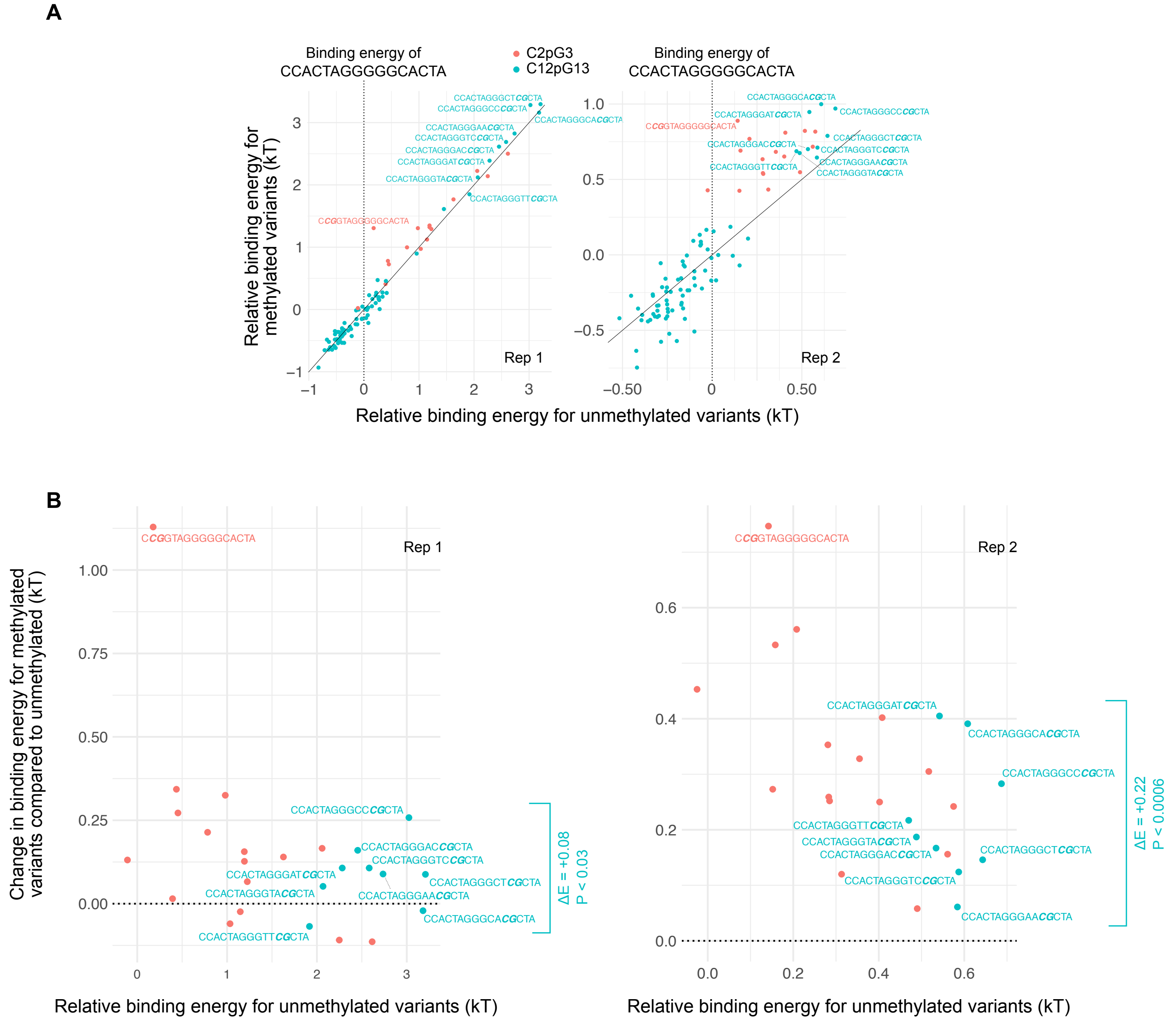

Fig S18. Effect of mC12pG13 methylation on *in vitro* CTCF binding. (**A**) Relative binding energy of CTCF toward unmethylated (x-axis) and methylated (y-axis) variants of its consensus binding sequence, obtained by Methyl-Spec-seq [28]. For each of the experimental replicates, only the variants that contain either a CpG in position 2/3 (red) or in position 12/13 (cyan) are shown. The variants that have a CpG in position 12/13 but lack the consensus G in the positions 10 and 11 are labeled. Also labeled in red is the variant CCGGTAGGGGGCACTA, which was previously found to have the largest sensitivity for C2pG3 methylation [28]. Binding energies are normalized to that of the consensus sequence CCACTAGGGGGCACTA, which was used in the original study for construction of other variants. Note that higher binding energy means lower affinity. (**B**) The y-axis shows the change in binding energy (ΔE) between unmethylated and methylated states of each sequence variant. Only the sequence variants are shown that either have a CpG in position 2/3 (red), or have a CpG in position 12/13 and lack G in positions 10 and 11 (cyan). For the latter category, a two-tailed t-test was used to test whether methylation of C12pG13 significantly affects the binding energy.
