# Supplementary material for: Toward a base-resolution panorama of the in vivo impact of cytosine methylation on transcription factor binding
Source: Genome Biol. 2022 Jul 7;23:151. doi: 10.1186/s13059-022-02713-y (PMC9264634; doi:10.1186/s13059-022-02713-y)
Supplement: Supplementary file 8 — Additional file 8:. Review history. [file 13059_2022_2713_MOESM8_ESM.docx]

Review History

**First round of review**

**Reviewer 1**

**Were you able to assess all statistics in the manuscript, including the appropriateness of statistical tests used?**

No, I do not feel adequately qualified to assess the statistics.

**Comments to author:**

In current work, Aldo Hernandez-Corchado and Hamed S. Najafabadi constructed a statistical model including parameters of TF sequence preference, genome accessibility, and regional methylation levels, and used that model trained from different samples to infer the in vivo impact of CpG methylation of TF binding signals. In the last years, as the high-throughput in vivo and in vitro technologies to characterize epigenetic effect of TF becomes mature, this field is being actively explored, so no doubt this is an important, timely work.

1) For CTCF, the authors reported positions 2 and 12 can block DNA interaction respectively. It is true previous work by John Staph group reported this through similar genomic analysis. However, Stormo group performed in vitro systematic scanning of CTCF to underlying sites and found only position 2 can block DNA binding, which is further confirmed by Xiaodong Cheng group's structural characterization work. Most certainly mCpG at position 12 has no physical effect on binding, though it doesn't necessarily mean absence of in vivo impact. My personal hypothesis is that the native sequence preference at position (12, 13) is CG, thus it exists prevalently in many in vivo sites, and serves as slightly better indicator of local methylation level than the one currently used, therefore from a statistical perspective, its 'in vivo impact' shows up as non-zero. It is difficult to assign the mCpG effect to some particular position when there are multiple CpGs within one binding site, so I suspect there are certain fractions of false discovery and suggest the authors be cautions to use the word "high precision" to describe current predicted results.

2) CTCF is the single most important genomic insulator which regulates the genome topology and accessibility for sure, so when you include local accessibility as parameter to predict its binding pattern, it should perform significantly better than most other TFs. More importantly, people generally agree that CTCF's binding pattern is achieved through a "Loop extrusion" process, instead of conventional equilibrium-based process. It is unfair and erroneous to use CTCF as the only example to demonstrate that "Differential TF binding across cell lines can be explained using JAMS models". There are some well-established cases of mCpG-sensitive TFs, e.g., Kaiso and ZFP57. It might be worthwhile to use them as positive controls to test the predictive power of JAMS model.

3) The mCpG impact is currently classified as plus, negative, no CpG, and no effect at Fig. 5. For many TFs of interest, the absence of CpG within the consensus sequence doesn't necessarily means it is insensitive to mCpG when there is CpG at some position, e.g., the position 2 of CTCF. Since the authors did large-scale statistical inference and only call those significant ones, "no effect" and "not currently found to have effect" are very different things. So, the authors should clarify these issues and fix them.

4) The C2H2-ZFs is composed of tandem array of zinc fingers and have some predicted motif through B1H recognition code, thus I suggest authors annotate the currently identified mCpG-sensitive positions with corresponding finger, which will help further validations by other specialists. For homodimer TFs like bHLH, bZIP and nuclear receptors , their symmetric configuration will make them sensitive to mCpG on each half site, which should also be annotated.

Overall, I think authors did very good analysis trying to infer the potential mCpG-sensitive positions of many TFs, but it is far from conclusive and complete to claim the results as "A base-resolution panorama". In many cases like KRAB-ZFPs, our incomplete understanding of about their motif structures and recognition modes significantly restrict the predictive powers of JAMS (Sup. Fig. 5), not to mention their methylation effect. Ideally, if authors of this work make certain novel predictions and reach out to geneticists or biophysicists for functional or biophysical validations about select TFs of interest, this would be a much better work and should have the potential to get published in very best place. I'm supportive the publication of this work, only if the authors acknowledge the practical limitation of statistical genomic analysis alone, make objective statement about the significance and contribution of the current work, and fix above mentioned issues.

**Reviewer 2**

**Were you able to assess all statistics in the manuscript, including the appropriateness of statistical tests used?**

Yes, and I have assessed the statistics in my report.

**Comments to author:**

In this paper, the authors use a generalized linear model (GLM) approach to predict ChIP-seq counts for a transcription factor (TF) of choice in terms of (i) the predicted affinity of the binding site a predicted from the underlying DNA sequence, (ii) the local accessibility of the chromatin as measured using ATAC-seq, and (iii) an position-specific effect of CpG methylation within the binding site. Letting these covariates compete with each other as predictors provides a way to break the confounding between methylation status and chromatin accessibility, which has made it difficult to infer the direct effect of CpG methylation on binding from ChIP-seq data.

This study is not the first to use a GLM approach to address this problem: Kribelbauer 2017 (PMID 28614722), not cited in the present manuscript, used a similar GLM methodology to infer the methylation sensitivity of p53 binding from ChIP-seq data while controlling for the confounding effect of chromatin accessibility. It would be interesting if the authors could comment on the conceptual and technical similarities and differences with their own approach. That said, the specific "JAMS" model presented here is novel, and this is the first comprehensive analysis across many TF's. As such, this is a study that should be of broad interest.

Detailed comments:

- page 4, line 10, "TF binding strength" : it may be confusing to readers to use this phrase to denote "occupancy" as opposed to "affinity". The former is the dependent variable in the GLM whereas the latter is one of the independent variable. To add to the confusion, in the Methods (page 18, line 32) the authors use the same phrase to refer to mu_f, the affinity. It would be good to be strict with definitions/wording.

- There are countless instance of statements about statistical significance that are not supported by a proper test and p-value. Example include "correlate strongly (page 4, line 57); still statistically significant (page 5, line 58); "significantly reduces" (page 6, line 36); "correlates negatively" (page 6, line 48); "strongly correlated" (page8, line 45).

- Page 7, line 57, "performance comparable": The word "comparable" seems somewhat misleading here, as the fraction of variance explained seems to be systematically lower when the model is not trained on the same cell line (up to 40% reduction in r^2). An F-test should confirm this. This reduction is expected, and because the correlation is still significant across cell lines, there is no reason to suggest that the models are perfectly "transferable" across cell lines. A slightly more sophisticated interpretation of the results would improve this section.

- Page 17, line 36, "CISP-BP" and "PFMs": should be CIS-BP (typo) and PFM (singular)

**Authors Response**

**Point-by-point responses to the reviewers’ comments:**

Reviewer #1:

In current work, Aldo Hernandez-Corchado and Hamed S. Najafabadi constructed a statistical model including parameters of TF sequence preference, genome accessibility, and regional methylation levels, and used that model trained from different samples to infer the in vivo impact of CpG methylation of TF binding signals. In the last years, as the high-throughput in vivo and in vitro technologies to characterize epigenetic effect of TF becomes mature, this field is being actively explored, so no doubt this is an important, timely work.

1) For CTCF, the authors reported positions 2 and 12 can block DNA interaction respectively. It is true previous work by John Staph group reported this through similar genomic analysis. However, Stormo group performed in vitro systematic scanning of CTCF to underlying sites and found only position 2 can block DNA binding, which is further confirmed by Xiaodong Cheng group's structural characterization work. Most certainly mCpG at position 12 has no physical effect on binding, though it doesn't necessarily mean absence of in vivo impact. My personal hypothesis is that the native sequence preference at position (12, 13) is CG, thus it exists prevalently in many in vivo sites, and serves as slightly better indicator of local methylation level than the one currently used, therefore from a statistical perspective, its 'in vivo impact' shows up as non-zero. It is difficult to assign the mCpG effect to some particular position when there are multiple CpGs within one binding site, so I suspect there are certain fractions of false discovery and suggest the authors be cautions to use the word "high precision" to describe current predicted results.

*Reply: We agree with the reviewer that the interpretation of mCpG effect at position 12 of the CTCF motif remains challenging, because methylation at this position can be highly correlated with methylation at position 2. To address the reviewer’s concern, we have included a paragraph in the discussion to ensure that the readers are aware of these challenges, especially for TFs with multiple CpGs in their binding sites:*

*Page 16: “We emphasize, however, that interpretation of intra-motif mCpG effects remains challenging for TFs that, similar to CTCF, recognize binding sequences with multiple CpGs. The methylation levels of such nearby CpGs are often highly correlated, which poses a substantial challenge for deconvolving the effect of methylation of each individual position. Also, when DNA accessibility data or regional methylation estimates are noisy, their confounding effect cannot be effectively decoupled from the true effect of intra-motif CpG methylation. In such cases, special attention needs to be given to the possibility of increased false positives in identifying intra-motif mCpG effects.”.*

*In addition, we have included new analyses and visualization to help with interpretation of our original observation regarding the in vivo effect of methylation of position 12. Specifically, we took advantage of the prevalence of CTCF genomic binding sites that do not have a CpG in position 2 but have a CpG at position 12, in order to show that mC12pG13 is significantly associated with decreased in vivo CTCF occupancy (Supplementary Fig. 15). We further noted that the Methyl-spec-seq dataset from Stormo group also contained a substantial number of binding site variants with CpA at position 2 and CpG at position 12; As we show in the new Supplementary Figure 16, it appears that when such variants have intermediate affinity for CTCF, methylation of C12pG13 increases the binding energy (and decreases the binding affinity) in vitro. Intriguingly, a very simple pattern, i.e. presence of G in either positions 10 or 11, explains the methyl-sensitivity of position 12: whenever both of these two positions lack the consensus G, methylation of C12pG13 significantly increases binding energy. These observations are now included and discussed in the paper, as we think they point to context-dependent methyl-sensitivity of position 12 in vitro, and provide additional information for interpreting our in vivo observed effects. This context-specificity may also explain why the effect of mC12pG13 was not detected by Cheng group, since their study used a DNA sequence with two G nucleotides in positions 10 and 11. Nonetheless, we recognize the limitations of our post hoc analysis of the data by Stormo group, and therefore emphasize in the Discussion the alternative explanations for the observed mC12pG13 effect.*

*Regarding the use of the phrase “high precision”, we would like to clarify that what we originally meant was precision in terms of identifying methyl-plus and methyl-minus TFs (i.e. the ratio of true positives to all predicted positive cases for each category, as now described on page 12). We have also modified the abstract to better reflect this issue. To see whether JAMS can precisely identify the intra-motif positions whose methylation can affect the TF binding, we performed a comparison of the mCpG effects found by JAMS and those revealed by bisulfite-SELEX (Yin et al., 2017). As the new Supplementary Figure 12 shows, in 93% of cases, the same position that shows a methylation effect based on JAMS analysis also shows a methylation effect based on bisulfite-SELEX data, suggesting that JAMS inferences are indeed precise with respect to the position that is affected by methylation. As mentioned above, we realize that in cases where the TF binding site contains multiple CpGs, delineating their individual effects can be difficult (and we now discuss this in the paper), but such cases do not seem to be prevalent as shown in Supplementary Figure 12 (and even for these multi-CpG motifs, 14 out 17 methyl-sensitive positions that are identified by JAMS are supported by bisulfite-SELEX).*

2) CTCF is the single most important genomic insulator which regulates the genome topology and accessibility for sure, so when you include local accessibility as parameter to predict its binding pattern, it should perform significantly better than most other TFs. More importantly, people generally agree that CTCF's binding pattern is achieved through a "Loop extrusion" process, instead of conventional equilibrium-based process. It is unfair and erroneous to use CTCF as the only example to demonstrate that "Differential TF binding across cell lines can be explained using JAMS models". There are some well-established cases of mCpG-sensitive TFs, e.g., Kaiso and ZFP57. It might be worthwhile to use them as positive controls to test the predictive power of JAMS model.

*Reply: We have now added differential binding analysis of CEBPB, MAX, and KAISO (ZBTB33); we selected these TFs based on their known sensitivity to CpG methylation and availability of ChIP-seq, DNase-seq and WGBS data for performing differential binding analysis across multiple cell lines. In all cases, we observed that the JAMS models of these TFs can successfully predict differential binding (median Pearson r=0.61, with >70% of cases showing r>0.5). The worst performance was observed for KAISO between HepG2 and K561 (r=0.16), but we note the higher performance of the model for predicting KAISO differential binding between GM12878 and HepG2 (r=0.44). When limiting the analysis to peaks that have similar accessibility across cell lines, intra-motif methylation alone was able to predict differential binding of these TFs to similar extent as (or better than) CTCF. These results are now included in Supplementary Figures 9-11.*

3) The mCpG impact is currently classified as plus, negative, no CpG, and no effect at Fig. 5. For many TFs of interest, the absence of CpG within the consensus sequence doesn't necessarily means it is insensitive to mCpG when there is CpG at some position, e.g., the position 2 of CTCF. Since the authors did large-scale statistical inference and only call those significant ones, "no effect" and "not currently found to have effect" are very different things. So, the authors should clarify these issues and fix them.

*Reply: We have now clarified this issue in the manuscript, providing explanation that “no effect” means no statistically significant effect found. We have kept the designation “no effect” in the figures for the sake of brevity but have added descriptions to the figure legends to prevent any misinterpretation. Specifically, both figures 4 and 5 include the sentence “Note that no-effect means no statistically significant effect was found”. Also, within the main text, we have modified the sentences to reflect the fact this issue. For example, on page 10: “we classified a TF as […] no-effect if the JAMS motif included a CpG but there was no statistically significant mCpG effect found by JAMS based on current data”.*

4) The C2H2-ZFs is composed of tandem array of zinc fingers and have some predicted motif through B1H recognition code, thus I suggest authors annotate the currently identified mCpG-sensitive positions with corresponding finger, which will help further validations by other specialists. For homodimer TFs like bHLH, bZIP and nuclear receptors , their symmetric configuration will make them sensitive to mCpG on each half site, which should also be annotated.

*Reply: As the reviewer suggested, we have now added annotation of zinc finger domains by comparing their recognition code predict motifs to the JAMS models. Supplementary Figure 7 graphically shows these annotations, which are also included in Supplementary Table 3. We have similarly added annotations of homodimer TFs, indicating whether the methyl-sensitive CpG is located in either of two motif-halves and/or at the motif center.*

Overall, I think authors did very good analysis trying to infer the potential mCpG-sensitive positions of many TFs, but it is far from conclusive and complete to claim the results as "A base-resolution panorama". In many cases like KRAB-ZFPs, our incomplete understanding of about their motif structures and recognition modes significantly restrict the predictive powers of JAMS (Sup. Fig. 5), not to mention their methylation effect. Ideally, if authors of this work make certain novel predictions and reach out to geneticists or biophysicists for functional or biophysical validations about select TFs of interest, this would be a much better work and should have the potential to get published in very best place. I'm supportive the publication of this work, only if the authors acknowledge the practical limitation of statistical genomic analysis alone, make objective statement about the significance and contribution of the current work, and fix above mentioned issues.

*Reply: We have now added a discussion of the limitations posed by KRAB-ZFPs on Page 13:*

*“We note that, overall, JAMS models were less predictive for KRAB-ZF proteins (Supplementary Fig. 5), potentially because for many of them a large fraction of the strongest binding sites overlap repetitive elements and, therefore, were excluded from our analyses. Thus, relatively fewer KRAB-ZF proteins were included in our high-confidence set of JAMS models, and the true fraction of methyl-plus KRAB-ZF proteins may be higher than our estimate.”*

*We also understand the reviewer’s concern about the phrasing of the title; we have now modified it to “Toward a base-resolution panorama of the in vivo impact of cytosine methylation on transcription factor binding”.*

*We thank the reviewer for their comments, and hope the manuscript revisions have addressed them.*

Reviewer #2:

In this paper, the authors use a generalized linear model (GLM) approach to predict ChIP-seq counts for a transcription factor (TF) of choice in terms of (i) the predicted affinity of the binding site a predicted from the underlying DNA sequence, (ii) the local accessibility of the chromatin as measured using ATAC-seq, and (iii) an position-specific effect of CpG methylation within the binding site. Letting these covariates compete with each other as predictors provides a way to break the confounding between methylation status and chromatin accessibility, which has made it difficult to infer the direct effect of CpG methylation on binding from ChIP-seq data.

This study is not the first to use a GLM approach to address this problem: Kribelbauer 2017 (PMID 28614722), not cited in the present manuscript, used a similar GLM methodology to infer the methylation sensitivity of p53 binding from ChIP-seq data while controlling for the confounding effect of chromatin accessibility. It would be interesting if the authors could comment on the conceptual and technical similarities and differences with their own approach. That said, the specific "JAMS" model presented here is novel, and this is the first comprehensive analysis across many TF's. As such, this is a study that should be of broad interest.

*Reply: We thank the reviewer for bringing the study be Kribelbauer et al. to our attention. As suggested, we have now included a brief discussion of the similarities and differences between JAMS and the GLM model used by Kribelbauer et al. (first paragraph of the Discussion section). Briefly, while both methods use a GLM to estimate the in vivo effect of methylation, their main differences include: (a) The GLM model presented by Kribelbauer et al. uses a binomial error family, while JAMS uses a negative binomial model that can take into account overdispersion; (b) the method used by Kribelbauer et al. requires the affinities of individual 10-mers to be provided as input (independent variables), while JAMS learns the coefficients that connect the sequence to in vivo TF occupancy; (c) JAMS takes into account DNA accessibility, which is not considered by Kribelbauer et al. We have also included a new Supplementary 14, which uses the example of CTCF to show the importance of considering DNA accessibility and using a GLM family that allows for overdispersion of read counts (overdispersion relative to the model-predicted mean values).*

Detailed comments:

- page 4, line 10, "TF binding strength" : it may be confusing to readers to use this phrase to denote "occupancy" as opposed to "affinity". The former is the dependent variable in the GLM whereas the latter is one of the independent variable. To add to the confusion, in the Methods (page 18, line 32) the authors use the same phrase to refer to mu_f, the affinity. It would be good to be strict with definitions/wording.

*Reply: This is an excellent point. We have now revised the text throughout the manuscript to use the phrase “TF occupancy” when appropriate. We also explicitly refer to mu_f as affinity.*

- There are countless instance of statements about statistical significance that are not supported by a proper test and p-value. Example include "correlate strongly (page 4, line 57); still statistically significant (page 5, line 58); "significantly reduces" (page 6, line 36); "correlates negatively" (page 6, line 48); "strongly correlated" (page8, line 45).

*Reply: We have now included measures of statistical significance when we make a related statement.*

- Page 7, line 57, "performance comparable": The word "comparable" seems somewhat misleading here, as the fraction of variance explained seems to be systematically lower when the model is not trained on the same cell line (up to 40% reduction in r^2). An F-test should confirm this. This reduction is expected, and because the correlation is still significant across cell lines, there is no reason to suggest that the models are perfectly "transferable" across cell lines. A slightly more sophisticated interpretation of the results would improve this section.

*Reply: We have now revised this section to avoid qualitative statements, and have tried to provide precise statements about the observed performances and their comparison across cell lines:*

*Page 7: “We observed that the CTCF JAMS model that was trained on HEK293 data could predict the ChIP-seq pulldown-to-control ratio in other cell types with a mean Pearson r of 0.62 and mean R2 of 0.38 (compared to 10-fold cross-validation r of 0.69 and R2 of 0.47 when applied to HEK293 data; Table 1).”*

- Page 17, line 36, "CISP-BP" and "PFMs": should be CIS-BP (typo) and PFM (singular)

*Reply: These typos are now fixed.*

*We thank the reviewer for their comments; we hope that our revisions have addressed the reviewer’s concerns.*

**Second round of review**

**Reviewer 1**

I appreciate that the authors took time to revise this paper, but still think the current version is oversell. The authors did large scale statistical analysis to infer the in vivo methylation impact upon TF binding by GLM approach. The maximal conclusion that could be drawn from this study is the positive/negative association of some intra-motif mCpG with TF occupancy, rather than the causal effect of mCpG upon TF binding, which are different things. Thus the value of this work depends on whether the predictions can be experimentally confirmed by others and whether those findings are proved functionally important in the future.

Due to the confounding effect of many uncontrolled factors, the predictive power of JAMS is very limited (Kaiso) and sometimes conflict with current in vitro results (position 12 at CTCF). Laying out the limitations in the main text should not be the primary reason to turn down a paper, otherwise "sweep negative results under the carpet" will become pervasive culture. Here are the detailed comments:

1) Due to above reasons, the statement in result section "revealing that 45% of TFs are inhibited by intra-motif methylation in vivo. In contrast, only 16 TFs (6%) preferentially bind to methylated sites" is inaccurate. "Revealing negative association of TF occupancy with intra-motif methylation in vivo for 45% of studied TFs, and 16 TFs are predicted to preferentially bind to methylated sites" should be the accepted form.

2) The conflicting finding of CTCF effect at position 12 with in vitro data should be mentioned in the CTCF results section; Similarly, the limited performance of Kaiso should also be mentioned in the main text explicitly.

3) It is true that many KRAB-ZFPs bind to repetitive elements, so our current knowledge about their motifs is far from complete, but even though we have complete knowledge about their motifs, it is still to be tested whether or not JAMS can effectively deconvolute their mCpG preference at base resolution. They are just too long. The senior author of this paper should know this very well. Pointing this out at main text before presenting Fig. 4 is important. Also, Kaiso has only 3 fingers and doesn't seem to have 15mer long motif. There are numerous papers about its methyl preference, e.g. PMID: 22949637, so some comparison might be worthwhile.

4) The term "High precision" in my mind means both high sensitivity and specificity, so does JAMS correctly detect all possible methyl-preferences for each studied TF? If not, I object to using that term and suggest using the term "set of high-confidence predictions" in results.

If above concerns are properly addressed by the authors, I suggest the editor considering accept this paper without further review.

**Reviewer 3**

In the current study, Hernandez-Corchado et al. use a General Linear Model with a negative-binomial error distribution (coined JAMS) to predict ChIP-seq occupancy for a large number of transcription factors (TFs) across several cell lines. The authors show that for a significant fraction of TFs the JAMS-inferred methylation preferences corresponds to those obtained by in-vitro studies, yet with a clear difference between methyl-minus and methyl-plus TFs, with the former achieving higher overall agreement. An interesting and relevant new finding is the identification of a group of C2H2(KRAB) ZF with a positive methylation effect and the use case of JAMS to improve the prediction of differential binding in new cell types.

The study marks a conceptual and technical improvement, in particular in scale, to previous studies. However, the challenges that arise when using steady-state ChIP-seq data to infer TF binding preferences for a dynamic mark demands special scrutiny, especially given the ambitious goal of providing a panorama of in vivo TF binding preferences. The request for a more careful discussion of potential confounders and limitations by reviewer #1 and #2 have generally been addressed in the first round of revisions, but require further clarification. The discrepancy in conformity with in vitro data between methyl-plus and methyl-minus TFs clearly indicate the presence of confounders, as there is no known mechanistic basis that could explain why TFs selectively abandon intrinsic binding preferences for one but not the other scenario. Although the authors provide a possible explanation - the competition with MBDs - experimental and modeling bias is not ruled out. Therefore, I recommend including additional controls that will improve the interpretability of the JAMS coefficients.

In particular, it is worth including an explicit discussion of the challenges that arise when modeling TF binding preference for the dynamically regulated 5mC mark from steady-state data. The 2017 study brought up by reviewer #2 (although not comparable in scale and execution) and a 2019 review by the same authors, bring up important points that are not sufficiently addressed in the revised version.

1) The authors point out that the 2017 study did not use accessibility. This is correct and a good point, yet neither the main text nor the methods specifically describe how accessibility was used in combination with regional methylation levels in the JAMS model. Is regional methylation derived from the 20 motif-flanking bps, or from the 10x larger window used for the accessibility indicator? This is important, as it is expected that regional methylation and accessibility are correlated and dependencies within 'independent' predictors may confound accurate effect size and directionality.

Would the coefficients change if only regional methylation levels or accessibility were used at a time? In the context of differential binding predictions, does the differential binding prediction rely on regional methylation or is it truly the local, intra-motif methylation status that is important? A clarification or an analysis similar to what has been done for accessibility would rule out this potential confounder.

2) Kribelbauer et al. use a peak-free approach to limit the bias that arises from ChIP-seq experiments tending to favor open regions and thus selectively eliminating reads from less accessible, yet likely more relevant regions (methylated regions tend to be closed and are thus more likely to be removed during size-selection). This is critical for methyl-plus TFs. The explanation provided by the authors for why a 'peak-free' version is not attempted is that affinities would be needed and that this is somehow worse than inferring the sequence preference directly from TF occupancy data. Both points are surprising, as the authors use in vitro derived binding models to 'align' their peaks and thus are perfectly capable of including relative affinities (PWM scores) directly in their model. An advantage of in vitro-derived non-5mCpG affinities as predictors is the removal of dependent variables that would otherwise need to be learned from potentially biased data, such as genomic sequence bias in open chromatin.

What if a similar peak-free approach was used, would this change the coefficients for methyl-plus TFs? Do base-specific indicators compete with the intra-motif methylation coefficients? Are sequence-specific coefficients for the full (plus methylation) and the reduced (no methylation) model comparable to each other, or are their suspicious changes, i.e. increased or decreased weights for TpG->CpG (T-mimickry)? In other words, are the sequence contexts between methylated and unmethylated motifs the same? A perhaps simple implementation would be to compare the current methylation coefficients to those derived from a model that directly uses the in vitro, non-methylated rel. binding affinities.

3) Another detail of the modeling framework that is not spelled out in the text are some implicit assumptions underlying the methylation predictors. Are the percentage methylated indicators directly used in the model? If so, how would the methylation coefficients change if methylation-status-ambiguous loci are successively removed from the analysis? In this context, ambiguous refers to genomic loci that comprise two distinct populations of cells that differ in their methylation status. For instance, a 50% methylation level means that half of the cells (more accurately, half of all alleles) had a 5mCpG, the other half did not. With the current data, there is unfortunately no way of knowing from which half the ChIP-seq signal is derived from and whether there is an underlying selection bias. Concretely, the JAMS model assumes that IP counts are uniformly drawn from the CpG and 5mCpG populations, which is an implicit, but questionable assumption (see i.e. single molecule footprinting data PMID: 33290745). In contrast, the 2017 study limits loci to those that have less than 20%, or more than 80% intra-motif methylation. Using 'pure' populations limits the potential confounder that ChIP-seq reads over proportionately stem from the more accessible, yet less-likely to be methylated fraction. This selection bias may trump intrinsic binding affinity and could explain the apparent discrepancy in methyl-minus and methyl-plus TFs. As a fairly easy-to-implement suggestion, the authors could visualize the change in 5mCpG-effect size when using increasingly stringent cutoffs on included loci (50-50, 40-60, 30-70, 20-80,10-90% methylation only, etc).

4) The 2017 study used TF-IP counts before and after drug-induced TF expression compared to IGG- or non-specific control reads. This differential setup, although still imperfect, is better suited to capture a TF's response to a dynamic mark compared to inference from steady state data alone. Evidently, such data does not exist for the vast majority of TFs, but the general limitation of steady-state inference should be discussed.

Minor points:

- As brought up by reviewer #2, binding affinity and occupancy are not always used appropriately. In the current version of the methods, TF affinity is used when referring to the response variable estimate. This is incorrect as TF affinity is an intrinsic property of the TF and does not depend on regional methylation, nor accessibility; TF occupancy does.

- To reiterate reviewer 1&2, potential conclusions need to put into an accurate context. In the discussion, it is mentioned that the methyl-plus TFs become indifferent to the methylation status. This cannot be concluded from the current models given the potential confounders listed above. Using 'apparent' should be applied consistently.

Overall, I agree with reviewers 1&2 that this study is the first comprehensive analysis of in vivo methylation binding preferences and thus support publication. The modeling framework, although not entirely novel, is well suited, improved and comprehensive compared to previous studies. However, I strongly recommend addressing the specific points above (especially point 3) for a more accurate and improved interpretation of in vivo TF methylation preferences.

**Authors Response**

**Point-by-point responses to the reviewers’ comments:**

Reviewer #1:

I appreciate that the authors took time to revise this paper, but still think the current version is oversell. The authors did large scale statistical analysis to infer the in vivo methylation impact upon TF binding by GLM approach. The maximal conclusion that could be drawn from this study is the positive/negative association of some intra-motif mCpG with TF occupancy, rather than the causal effect of mCpG upon TF binding, which are different things. Thus the value of this work depends on whether the predictions can be experimentally confirmed by others and whether those findings are proved functionally important in the future.

Due to the confounding effect of many uncontrolled factors, the predictive power of JAMS is very limited (Kaiso) and sometimes conflict with current in vitro results (position 12 at CTCF). Laying out the limitations in the main text should not be the primary reason to turn down a paper, otherwise "sweep negative results under the carpet" will become pervasive culture. Here are the detailed comments:

1) Due to above reasons, the statement in result section "revealing that 45% of TFs are inhibited by intra-motif methylation in vivo. In contrast, only 16 TFs (6%) preferentially bind to methylated sites" is inaccurate. "Revealing negative association of TF occupancy with intra-motif methylation in vivo for 45% of studied TFs, and 16 TFs are predicted to preferentially bind to methylated sites" should be the accepted form.

*Reply: We have now modified the abstract to reflect the reviewer’s suggestion:*

*“[…] revealing a negative association between in vivo TF occupancy and intra-motif methylation for 45% of studied TFs, as well as 16 TFs that are predicted to bind to methylated sites […]”*

2) The conflicting finding of CTCF effect at position 12 with in vitro data should be mentioned in the CTCF results section; Similarly, the limited performance of Kaiso should also be mentioned in the main text explicitly.

*Reply: As requested by the reviewer, we have now added these points in the Results section.*

*Page 6-7: “We note, however, that the predicted effect of methylation of C12pG13 is not currently supported by in vitro data (see Discussion).”*

*Page 12: “However, we observed a comparably limited performance for predicting the differential binding of KAISO (Pearson correlation between 0.16 and 0.44 for KAISO differential binding across different cell lines, compared to median Pearson correlation of 0.61 for CEBP and MAX).”*

3) It is true that many KRAB-ZFPs bind to repetitive elements, so our current knowledge about their motifs is far from complete, but even though we have complete knowledge about their motifs, it is still to be tested whether or not JAMS can effectively deconvolute their mCpG preference at base resolution. They are just too long. The senior author of this paper should know this very well. Pointing this out at main text before presenting Fig. 4 is important. Also, Kaiso has only 3 fingers and doesn't seem to have 15mer long motif. There are numerous papers about its methyl preference, e.g. PMID: 22949637, so some comparison might be worthwhile.

*Reply: To address the reviewer’s concern about KRAB-ZFPs, we now explicitly discuss the potential problems caused by their unusually long motifs:*

*Page 17: “The in vivo methylation levels of such nearby CpGs are often highly correlated, which poses a substantial challenge for deconvolving the effect of methylation of each individual position. This is particularly the case for multi-zinc finger proteins such as the KRAB-ZF family, whose binding motifs are often longer than other TF families. Such long binding sites may impose additional difficulties for deconvolving the effect of methylation of individual intra-motif CpGs, and it remains to be tested whether JAMS inferences for this family have base pair resolution.”*

*Regarding the motif identified for KAISO, we would like to point out that many other sources also report a motif that is longer than the 9mer expected from the zinc fingers alone. For example, using Methyl-SELEX, Yin et al. (2017) identified a motif that is 20bp long, and various ChIP-seq motif analyses have identified sequences that are ~15bp long, although the motif information content is not uniform across the 15bp region (e.g., see https://hocomoco11.autosome.org/motif/KAISO_HUMAN.H11MO.0.A). We note, however, that the motifs from these sources appear to have a common CGCG core, which also appears in our motif and is the region whose methylation increases the affinity of KAISO.*

4) The term "High precision" in my mind means both high sensitivity and specificity, so does JAMS correctly detect all possible methyl-preferences for each studied TF? If not, I object to using that term and suggest using the term "set of high-confidence predictions" in results.

*Reply: We agree with the reviewer that, given the common meaning of the term “precision”, this term may create confusion. To prevent any misinterpretation, we have now replaced the term “precision” with the term “positive predictive value”, which has the same meaning in classification problems but is less ambiguous:*

*In the abstract: “We show that JAMS models […] have high positive predictive value for identification of TFs affected by intra-motif methylation”.*

*Also on page 12: “precision or positive predictive value: ratio of true positives to all predicted positive cases”.*

If above concerns are properly addressed by the authors, I suggest the editor considering accept this paper without further review.

*Reply: We appreciate the time the reviewer took to assess our work and provide feedback. We hope the revised version of the manuscript has addressed the reviewer’s remaining concerns.*

Reviewer #3:

In the current study, Hernandez-Corchado et al. use a General Linear Model with a negative-binomial error distribution (coined JAMS) to predict ChIP-seq occupancy for a large number of transcription factors (TFs) across several cell lines. The authors show that for a significant fraction of TFs the JAMS-inferred methylation preferences corresponds to those obtained by in-vitro studies, yet with a clear difference between methyl-minus and methyl-plus TFs, with the former achieving higher overall agreement. An interesting and relevant new finding is the identification of a group of C2H2(KRAB) ZF with a positive methylation effect and the use case of JAMS to improve the prediction of differential binding in new cell types.

The study marks a conceptual and technical improvement, in particular in scale, to previous studies. However, the challenges that arise when using steady-state ChIP-seq data to infer TF binding preferences for a dynamic mark demands special scrutiny, especially given the ambitious goal of providing a panorama of in vivo TF binding preferences. The request for a more careful discussion of potential confounders and limitations by reviewer #1 and #2 have generally been addressed in the first round of revisions, but require further clarification. The discrepancy in conformity with in vitro data between methyl-plus and methyl-minus TFs clearly indicate the presence of confounders, as there is no known mechanistic basis that could explain why TFs selectively abandon intrinsic binding preferences for one but not the other scenario. Although the authors provide a possible explanation - the competition with MBDs - experimental and modeling bias is not ruled out. Therefore, I recommend including additional controls that will improve the interpretability of the JAMS coefficients.

In particular, it is worth including an explicit discussion of the challenges that arise when modeling TF binding preference for the dynamically regulated 5mC mark from steady-state data. The 2017 study brought up by reviewer #2 (although not comparable in scale and execution) and a 2019 review by the same authors, bring up important points that are not sufficiently addressed in the revised version.

1) The authors point out that the 2017 study did not use accessibility. This is correct and a good point, yet neither the main text nor the methods specifically describe how accessibility was used in combination with regional methylation levels in the JAMS model. Is regional methylation derived from the 20 motif-flanking bps, or from the 10x larger window used for the accessibility indicator? This is important, as it is expected that regional methylation and accessibility are correlated and dependencies within 'independent' predictors may confound accurate effect size and directionality.

*Reply: We thank the reviewer for pointing out our omission to explicitly describe how regional methylation is calculated and used in the model. We have now added more details to the Methods section to clarify this point:*

*On page 19, we have added: “the average regional methylation and base composition in the flanking 20-bp regions were used as covariates in the model”.*

*On page 20: “These m features include those related to accessibility (A), intra-motif methylation (M), sequence (S), regional sequence composition (RS), and regional methylation (RM): X=[X_A, X_M, X_S, X_RS, X_RM]”.*

*Calculation of the regional methylation from the 20bp flanking regions is also indicated schematically in Figure 1C.*

Would the coefficients change if only regional methylation levels or accessibility were used at a time? In the context of differential binding predictions, does the differential binding prediction rely on regional methylation or is it truly the local, intra-motif methylation status that is important? A clarification or an analysis similar to what has been done for accessibility would rule out this potential confounder.

*Reply: We have now added a new panel to Supplementary Figure 16 (panel B), which shows how intra-motif methylation coefficients for CTCF change if regional methylation or accessibility were removed from the model. As Figure S16B shows, removing accessibility has a profound effect on the estimation of intra-motif methylation effects. Removing regional methylation levels has a much smaller effect, potentially because most of its confounding effect is already captured by accessibility. These analyses further support our previous observations that including accessibility in the model is crucial for estimation of intra-motif methylation effects.*

*As the reviewer suggested, to show that the differential binding can be predicted by our model independent of regional methylation, we have added a new analysis, which is shown in the new Supplementary Figure 6. Specifically, we focused on the CTCF peaks that, in addition to having similar accessibility between two cell lines, do not have any CpGs in their flanking regions (in order to avoid the confounding effect of such flanking CpGs). Figure S6 shows that the CTCF JAMS model is still able to predict the differential binding of these peaks, which should be driven by the intra-motif methylation levels given that there are no flanking CpGs and no differences in accessibility.*

2) Kribelbauer et al. use a peak-free approach to limit the bias that arises from ChIP-seq experiments tending to favor open regions and thus selectively eliminating reads from less accessible, yet likely more relevant regions (methylated regions tend to be closed and are thus more likely to be removed during size-selection). This is critical for methyl-plus TFs. The explanation provided by the authors for why a 'peak-free' version is not attempted is that affinities would be needed and that this is somehow worse than inferring the sequence preference directly from TF occupancy data. Both points are surprising, as the authors use in vitro derived binding models to 'align' their peaks and thus are perfectly capable of including relative affinities (PWM scores) directly in their model. An advantage of in vitro-derived non-5mCpG affinities as predictors is the removal of dependent variables that would otherwise need to be learned from potentially biased data, such as genomic sequence bias in open chromatin.

*Reply: We believe the reviewer is referring to the Methods section where we write “We used this permissive p-value threshold [of 0.01] to obtain a range of TF binding signals, which our method uses to quantitatively model TF occupancy”. We would like to emphasize that in fact we intentionally included peaks with very low ChIP-seq signal (given the permissive p-value threshold of 0.01 for peak calling), as well as regions that have a high background signal and potentially no TF binding signal (by inversing the pull-down and control experiments), as described in the Methods section. This means that the genomic regions that are excluded from our analysis are only those that have almost no pull-down signal and no background signal, which are likely not informative for learning the determinants of TF occupancy. This is, in spirit, similar to the approach used by Kribelbauer et al., where they kept “only motifs with >=1x coverage in both control and IP”.*

*In addition, we would like to clarify that using an entirely peak-free approach would not be compatible with JAMS model specification. As the reviewer has noted, we use the seed PWMs to find the “best” position within each locus, but we should note that many loci, particularly those with low or no TF signal, have no strong PWM matches. Therefore, the best PWM hit in these loci is essentially a random-looking position with very low similarity to the seed PWM. This variability in sequence across the loci is the key for JAMS ability to learn the sequence determinants of ChIP-seq-based TF occupancy. However, this strategy cannot be extended to the whole genome: if we searched the entire genome for hits that highly match the seed PWM (similar to Kribelbauer et al.), there would essentially be no or very little variability in the sequences of these hits, preventing JAMS from learning the sequence-specific coefficients (motif logos). In addition, some of the strong ChIP-seq peaks may not include any PWM hits, which would be excluded from this analysis and, therefore, their information regarding accessibility and/or regional methylation level would not be used by the model. An alternative is to include both high- and low-scoring PWM hits genome-wide, but this requires inclusion of tens of millions of sequences in model fitting, which poses substantial computational challenges.*

What if a similar peak-free approach was used, would this change the coefficients for methyl-plus TFs? Do base-specific indicators compete with the intra-motif methylation coefficients? Are sequence-specific coefficients for the full (plus methylation) and the reduced (no methylation) model comparable to each other, or are their suspicious changes, i.e. increased or decreased weights for TpG->CpG (T-mimickry)? In other words, are the sequence contexts between methylated and unmethylated motifs the same? A perhaps simple implementation would be to compare the current methylation coefficients to those derived from a model that directly uses the in vitro, non-methylated rel. binding affinities.

*Reply: As discussed above, a peak-free approach is incompatible with JAMS model specification and would not allow us to learn the sequence-specific coefficients. However, we have followed the reviewer’s suggestion to compare the CTCF sequence-specific coefficients between the full model and a reduced model with no intra-motif methylation. As the new Supplementary Figure 1 shows, there are almost no changes in the sequence-specific coefficients.*

*We are not sure about the exact meaning of the suggestion by the reviewer to compare the current methylation coefficients to those derived from a model that uses non-methylated relative binding affinities (since methylation coefficients cannot be derived from non-methylated sequences). If the reviewer means comparing sequence-specific coefficients for the JAMS model that considers methylation, vs. motif models learned from in vitro data, we would like to point out that this is in fact part of our quality control for identifying high-confidence models, as mentioned on page 10: “[…] we selected at most one representative model per TF based on the following criteria: […] (iii) Pearson correlation >0.3 between the known and JAMS-inferred sequence motif”.*

3) Another detail of the modeling framework that is not spelled out in the text are some implicit assumptions underlying the methylation predictors. Are the percentage methylated indicators directly used in the model? If so, how would the methylation coefficients change if methylation-status-ambiguous loci are successively removed from the analysis? In this context, ambiguous refers to genomic loci that comprise two distinct populations of cells that differ in their methylation status. For instance, a 50% methylation level means that half of the cells (more accurately, half of all alleles) had a 5mCpG, the other half did not. With the current data, there is unfortunately no way of knowing from which half the ChIP-seq signal is derived from and whether there is an underlying selection bias. Concretely, the JAMS model assumes that IP counts are uniformly drawn from the CpG and 5mCpG populations, which is an implicit, but questionable assumption (see i.e. single molecule footprinting data PMID: 33290745). In contrast, the 2017 study limits loci to those that have less than 20%, or more than 80% intra-motif methylation. Using 'pure' populations limits the potential confounder that ChIP-seq reads over proportionately stem from the more accessible, yet less-likely to be methylated fraction. This selection bias may trump intrinsic binding affinity and could explain the apparent discrepancy in methyl-minus and methyl-plus TFs. As a fairly easy-to-implement suggestion, the authors could visualize the change in 5mCpG-effect size when using increasingly stringent cutoffs on included loci (50-50, 40-60, 30-70, 20-80,10-90% methylation only, etc).

*Reply: We have now added a sentence to the Methods section to explicitly indicate how we use methylation in our model:*

*Page 19: “the fraction of methylated reads per position was directly used in the model”.*

*Also, we have followed the reviewer’s suggestion and examined the effect size of mCpG on CTCF binding after removing ambiguous loci with increasingly stringent cutoffs. As the new panel C in Supplementary Figure 2 shows, filtering out ambiguous loci has almost no effect on the estimated effect sizes; we only start to lose statistical power at the most stringent cutoff (10-90) because a relatively large fraction of loci is removed.*

4) The 2017 study used TF-IP counts before and after drug-induced TF expression compared to IGG- or non-specific control reads. This differential setup, although still imperfect, is better suited to capture a TF's response to a dynamic mark compared to inference from steady state data alone. Evidently, such data does not exist for the vast majority of TFs, but the general limitation of steady-state inference should be discussed.

*Reply: We thank the reviewer for pointing this out; we have now added the potential drawbacks of using steady-state TF occupancy data to the discussion:*

*Page 17: “In addition, reliance on steady-state TF occupancy data poses additional challenges for correctly modeling the determinants of in vivo TF occupancy, especially for low-affinity binding sites [35]. While the vast majority of available datasets represent the steady-state binding profiles of TFs, modeling how TF occupancy changes after in vivo modulation of TF concentration may provide a more nuanced view of the determinants of TF specificity [34, 35].”*

Minor points:

- As brought up by reviewer #2, binding affinity and occupancy are not always used appropriately. In the current version of the methods, TF affinity is used when referring to the response variable estimate. This is incorrect as TF affinity is an intrinsic property of the TF and does not depend on regional methylation, nor accessibility; TF occupancy does.

*Reply: We have now modified the text in the Methods section (page 20) to avoid incorrect use of affinity.*

- To reiterate reviewer 1&2, potential conclusions need to put into an accurate context. In the discussion, it is mentioned that the methyl-plus TFs become indifferent to the methylation status. This cannot be concluded from the current models given the potential confounders listed above. Using 'apparent' should be applied consistently.

*Reply: As the reviewer has suggested, we now use the term “apparent methyl-indifferent”. For example, on page 15: “[…] we have identified a total of 67 apparent methyl-indifferent TFs in vivo”.*

Overall, I agree with reviewers 1&2 that this study is the first comprehensive analysis of in vivo methylation binding preferences and thus support publication. The modeling framework, although not entirely novel, is well suited, improved and comprehensive compared to previous studies. However, I strongly recommend addressing the specific points above (especially point 3) for a more accurate and improved interpretation of in vivo TF methylation preferences.

*Reply: We thank the reviewer for supporting the publication of our work. We hope that our revisions have addressed the points raised by the reviewer.*

**Third round of review**

**Reviewer 1**

My concerns in previous reviews have been addressed.

One remaining issue is, when I opened the Github repo for JAMS model, the only available data analysis workflow is for CTCF. To ensure the reproducibility of this work, the analysis workflow for all tested TFs, or at least those mentioned ones in the main text, e.g., KAISO, should be openly available.

Hope the major findings will be proved correct in the future and shine new lights on this field.

**Reviewer 3**

The authors addressed my main points of concern. The suggested controls were added. It is reassuring to see that JAMS model parameters are robust even while iteratively adding loci with distinct methylation statuses (i.e. 50% methylated 50% not). Personally, I would have liked to see this analysis extended to more TFs, not just CTCF. Given CTCF's specific role as a bookmarking factor and with stable binding across many cell types, CTCF is perhaps less likely to be susceptible to biased sampling. I.e. ChIP-seq reads being preferentially drawn from more accessible, less methylated regions. Methylation effects for TFs with shorter residence times/inability to footprint, might be more susceptible to a potential sampling bias.

Since the authors adjusted the main text, discussing the limitations and unknown parameters that may impact inference of methylation preferences in vivo, it is however not strictly necessary to include more controls. I therefore recommend publication without further review.

**Authors Response**

**Point-by-point responses to the reviewers’ comments:**

Reviewer 1#:

My concerns in previous reviews have been addressed.

One remaining issue is, when I opened the Github repo for JAMS model, the only available data analysis workflow is for CTCF. To ensure the reproducibility of this work, the analysis workflow for all tested TFs, or at least those mentioned ones in the main text, e.g., KAISO, should be openly available.

Hope the major findings will be proved correct in the future and shine new lights on this field.

*Reply: The workflow for reproducing the JAMS models have been added to the GitHub repository under the “run_JAMS_for_manuscript_TFs” directory. The corresponding input data used by JAMS are also added to Zenodo for all the 260 TFs described in the manuscript (the input peaks and output JAMS models for all the samples can be found in Zenodo as compressed package files “macs_peaks.tar.gz” and “all_JAMS_models_text.tar.gz”).*
